# Supplementary material for: Newcastle disease burden in Nepal and efficacy of Tablet I2 vaccine in commercial and backyard poultry production
Source: PLoS One. 2023 Mar 10;18(3):e0280688. doi: 10.1371/journal.pone.0280688 (PMC10004539; doi:10.1371/journal.pone.0280688)
Supplement: S1 File — (DOCX) [file pone.0280688.s001.docx]

**Supplementary Data (Table)**

**Table S 1**: NDV F gene reference sequence was taken from the NCBI GenBank representing various genotypes of NDV.

| **Accession no.** | **Genotype** | **Host** | **Country** |
| --- | --- | --- | --- |
| AF217084 | I | Vaccinal | NA |
| AY562991 | I | Vaccinal | UK |
| AY935499 | I | Vaccinal | NA |
| AY965079 | I | Duck | Russia |
| DQ097394 | I | Vaccinal | NA |
| EF464163 | I | Swine | China |
| GQ918280 | I | Black-headed gull | Sweden |
| JQ029740 | I | Duck | China |
| JQ966080 | I | Ring-necked pheasant | South Korea |
| KC503453 | I | American green winged teal | USA |
| KC503476 | I | Northern pintail | USA |
| KC808493 | I | Red-lored amazon parrot | Mexico |
| KM056356 | I | Chicken | India |
| MH996911 | I | Chicken | Nigeria |
| MK204384 | I | Red necked stint | Australia |
| AF309418 | II | Vaccinal | NA |
| AY289002 | II | Turkey | USA |
| DQ195265 | II | Vaccinal | USA |
| EU289028 | II | Vaccinal | NA |
| EU330230 | II | Chicken | India |
| FJ480823 | II | Goose | China |
| GU978777 | II | Chicken | USA |
| JN872151 | II | Chicken | USA |
| JN942019 | II | Avian | Peru |
| JX316216 | II | Chicken | India |
| KC987036 | II | Chicken | India |
| KU133356 | II | Peregrine falcon | Brazil |
| KU133362 | II | Pigeon | Ukraine |
| KU159667 | II | Rock pigeon | USA |
| KU200238 | II | Chicken | China |
| KU527558 | II | Pigeon | China |
| KY788664 | II | Cattle egret | China |
| KY788670 | II | Common moorhen | China |
| MG686584 | II | Duck | Pakistan |
| MH996914 | II | Vulture | Nigeria |
| MH996916 | II | River eagle | Nigeria |
| MH996947 | II | Chicken | Nigeria |
| Y18898 | II | Vaccinal | NA |
| EF201805 | III | Avian | China |
| GU182327 | III | Chicken | Pakistan |
| MH996904 | III | Pigeon | Bulgaria |
| AY741404 | IV | Fowl | UK |
| M24702 | IV | Chicken | Japan |
| MH996900 | IV | Pullet | Bulgaria |
| EU518684 | V | Chicken | Mexico |
| JN872189 | V | Parrot | USA |
| JN942027 | V | Fighting cock | Nicaragua |
| FJ410145 | VI | Pigeon | USA |
| FJ865434 | VI | Pigeon | China |
| HG326604 | VI | Chicken | Nigeria |
| JX094510 | VI | Pigeon | China |
| JX901124 | VI | Pigeon | Belgium |
| AY028995 | VII | Fowl | China |
| DQ227246 | VII | Goose | China |
| KU862293 | VII | Parakeet | Pakistan |
| KX496967 | VII | Pigeon | Pakistan |
| MF622047 | VII | Chicken | South Africa |
| MH891651 | VII | Green-winged teal | Pakistan |
| AF048763 | VIII | Chicken | Malaysia |
| AY734534 | VIII | Chicken | Argentina |
| FJ751919 | VIII | Chicken | China |
| FJ705468 | X | Mottled duck | USA |
| KX857721 | X | Mallard | USA |
| HQ266602 | XI | Chicken | Madagascar |
| JX518884 | XI | Chicken | Madagascar |
| JN627507 | XII | Goose | China |
| KU594615 | XII | Chicken | Peru |
| KU594618 | XII | Chicken | Peru |
| GU182323 | XIII | Chicken | Pakistan |
| GU585905 | XIII | Chicken | Sweden |
| JN942034 | XIII | Ostrich | South Africa |
| KT734767 | XIII | Chicken | India |
| HF969187 | XIV | Chicken | Nigeria |
| JN872165 | XIV | Chicken | USA |
| JQ039386 | XIV | Chicken | Nigeria |
| FJ705456 | XIX | Cormorant | USA |
| JN942024 | XIX | Cormorant | USA |
| FJ772455 | XVIII | Avian | Mauritania |
| HF969218 | XVIII | Pigeon | Ivory Coast |
| JX518886 | XVIII | Chicken | Mali |
| AB853928 | XX | Chicken | Japan |
| AF458016 | XX | Chicken | China |
| KY042142 | XX | Quail | South Korea |
| KU377535 | XXI | Turtledove | Italy |
| KU862298 | XXI | Pigeon | Pakistan |
| KY042132 | XXI | Pigeon | Egypt |
| MZ087886 | II | Chicken | Nepal |
| MZ087890 | II | Chicken | Nepal |
| MZ087885 | I | Chicken | Nepal |
| NA | I | Chicken | Nepal |
| MZ087888 | II | Chicken | Nepal |
| MZ087887 | II | Chicken | Nepal |
| MZ087889 | II | Chicken | Nepal |

**Table S 2**: Ranigoldunga^TM^ I-2 ND Vaccine- In-vivo Trial.

| **RanigoldungaTM I-2 ND Vaccine- In-vivo Trial** | | | | | | | | | |
| --- | --- | --- | --- | --- | --- | --- | --- | --- | --- |
|  |  |  |  |  |  |  |  |  |  |
|  |  | Antibody Titre | | | | | | | |
| Formulation | Chicken ID | Week 0 | Week 1 | Week 2 | Week 3 | Week 4 | Week 5 | Week 6 | Week 7 |
| Negative Control | 1 | 174.1406695 | 116.0937796 | 147.7557195 | 21.10795994 | 15.83096995 | 79.15484976 | 268.385632 | 56.43658989 |
|  | 2 | 10.55397997 | 42.21591987 | 253.2955192 | 353.630179 | 110.854968 | 63.32387981 | 121.3707696 | 98.25479659 |
|  | 3 | 52.76989984 | 79.15484976 | 295.5114391 | 110.8167897 | 161.697865 | 116.0937796 | 94.98581971 | 110.36984 |
|  | 4 | 10.55397997 | 21.10795994 | 142.4787296 | 26.38494992 | 10.55397997 | 153.0327095 | 248.0185292 | 254.3695246 |
|  | 5 | 153.0327095 | 42.21591987 | 184.6946494 | 63.32387981 | 156.712045 | 147.7557195 | 464.3751186 | 365.474582 |
|  | 6 | 52.76989984 | 42.21591987 | 131.9247496 | 179.4176594 | 153.650975 | 174.1406695 | 195.2486294 | 138.2654799 |
| Tablet Formulation | 7 | 464.3751186 | 1588.373985 | 3123.97807 | 2976.222351 | 3678.062019 | 3108.1471 | 1878.608434 |  |
|  | 8 | 195.2486294 | 1598.927965 | 2474.908302 | 4047.451318 | 5118.680284 | 4949.816605 | 3509.198339 |  |
|  | 9 | 94.98581971 | 1551.435055 | 2226.889773 | 2601.556062 | 1535.604085 | 1467.003215 | 1076.505957 |  |
|  | 10 | 52.76989984 | 174.1406695 | 2786.250711 | 4047.451318 | 3282.28777 | 3028.992251 | 2496.016262 |  |
|  | 11 | 89.70882972 | 110.8167897 | 3118.70108 | 3044.823221 | 2849.574591 | 2145.08603 | 1846.946494 |  |
|  | 12 | 163.5866895 | 232.1875593 | 1994.702214 | 3013.161281 | 1741.406695 | 1274.382683 | 1229.538666 |  |
| Lyophilized formulation | 13 | 47.49290985 | 105.5397997 | 195.2486294 | 1546.158065 | 1319.247496 | 1197.876726 |  | 1445.895256 |
|  | 14 | 21.10795994 | 163.5866895 | 1899.716394 | 4865.384765 | 3683.339009 | 2459.077332 |  | 2126.626963 |
|  | 15 | 42.21591987 | 195.2486294 | 437.9901687 | 2015.810174 | 2237.443753 | 1456.449236 |  | 1052.749104 |
|  | 16 | 47.49290985 | 712.3936478 | 1936.655324 | 3155.64001 | 3683.339009 | 3023.715261 |  | 2110.795994 |
|  | 17 | 52.76989984 | 453.8211386 | 1213.707696 | 2596.279072 | 2965.668371 | 1963.040274 |  | 1583.096995 |
|  | 18 | 79.15484976 | 944.5812071 | 891.8113073 | 2195.227833 | 2058.026094 | 2232.166763 |  | 2184.936663 |
| Liquid Formulation | 19 | 21.10795994 | 137.2017396 | 1345.632446 | 3778.324828 | 4327.131787 | 2765.142752 |  | 2406.307433 |
|  | 20 | 89.70882972 | 147.7557195 | 680.7317079 | 2680.710912 | 1461.726226 | 1155.660806 |  | 1081.782947 |
|  | 21 | 26.38494992 | 221.6335793 | 1551.435055 | 3984.127438 | 4332.408777 | 3783.601818 |  | 2875.959541 |
|  | 22 | 21.10795994 | 395.7742488 | 712.3936478 | 1984.148234 | 1973.594254 | 2401.030443 |  | 1514.496125 |
|  | 23 | 63.32387981 | 121.3707696 | 506.5910384 | 3034.269241 | 3823.715261 | 2601.556062 |  | 1201.69379 |
|  | 24 | 58.04688982 | 89.70882972 | 934.0272271 | 3936.634528 | 3087.039141 | 2401.030443 |  | 2196.804691 |

**Table S 3**: Antibody level against NDV of different test groups monitored before challenge trial with Genotype VIIc.

| Particulars | | Week 3 | | Week 4 | |
| --- | --- | --- | --- | --- | --- |
| Test Group | Chicken ID | Titre | Result | Titre | Result |
| Vaccinated | 1 | 491.3292 | Negative | 4669.692 | Positive |
| Vaccinated | 2 | 4554.085 | Positive | 9236.164 | Positive |
| Vaccinated | 3 | 1531.791 | Positive | 5388.106 | Positive |
| Vaccinated | 4 | 1845.581 | Positive | 7014.86 | Positive |
| Vaccinated | 5 | 1952.93 | Positive | 5503.713 | Positive |
| Vaccinated | 6 | 2828.24 | Positive | 6890.996 | Positive |
| Vaccinated | 7 | 4347.644 | Positive | 7848.881 | Positive |
| Vaccinated | 8 | 4405.448 | Positive | 7394.711 | Positive |
| Vaccinated | 9 | 2613.541 | Positive | 7857.139 | Positive |
| Vaccinated | 10 | 1573.079 | Positive | 4958.709 | Positive |
| Vaccinated | 11 | 1622.625 | Positive | 4620.146 | Positive |
| Vaccinated | 12 | 499.5869 | Negative | 2588.768 | Positive |
| Vaccinated | 13 | 1011.56 | Positive | 2621.799 | Positive |
| Vaccinated | 14 | 2093.31 | Positive | 5231.211 | Positive |
| Control | 15 | 86.70516 | Negative | 103.2204 | Negative |
| Control | 16 | 152.7662 | Negative | 78.44752 | Negative |
| Control | 17 | #NUM! | #NUM! | 53.67462 | Negative |
| Control | 18 | 12.38645 | Negative | 86.70516 | Negative |
| Control | 19 | 590.4208 | Negative | #NUM! | #NUM! |
| Control | 20 | 28.90172 | Negative | 152.7662 | Negative |
| Control | 21 | 53.67462 | Negative | 94.96279 | Negative |
| Control | 22 | #NUM! | #NUM! | 28.90172 | Negative |
| Control | 23 | 119.7357 | Negative | 103.2204 | Negative |
| Control | 24 | 78.44752 | Negative | 78.44752 | Negative |
| Control | 25 | #NUM! | #NUM! | 20.64408 | Negative |
| Control | 26 | 4.128817 | Negative | 565.6479 | Negative |
| Control | 27 | 590.4208 | Negative | 94.96279 | Negative |

**Table S 4:** Stability test of Ranigoldhunga^TM^ stain I2 at different temperature (4°C, 37°C) and ambient temperature monitored till EID_50_/ml of >6 per ml.

| Days of Storage | EID_50_ /ml value | | |
| --- | --- | --- | --- |
|  | Ambient | 4°C | 37°C |
| 0 | 7.9 | 8.0 | 7.5 |
| 4 | 7.8 | 7.9 | 7.0 |
| 7 | 7.7 | 7.9 | 6.6 |
| 10 | 7.6 | 7.9 | 6.3 |
| 14 | 7.6 | 7.9 | 5.7 |
| 28 | 7.3 | 7.8 | 3.9 |
| 60 | 6.6 | 7.5 |  |
| 90 | 5.9 | 7.3 |  |
| 120 | 5.3 | 7.1 |  |
| 150 | 4.7 | 6.9 |  |
| 180 | 4.0 | 6.7 |  |

**Table S 5:** HI titres (log 2) of chickens in Goldhunga farm-vaccinated through Ocular (tablet) method.

| Goldhunga Ocular | | | |
| --- | --- | --- | --- |
| HI titers (log 2) | % of chickens | % of 2^3 and above HI titer | % of 2^4 and above HI titer |
| 0 | 0 | 89 | 78 |
| 1 | 2.9 |  |  |
| 2 | 8.3 |  |  |
| 3 | 10.8 |  |  |
| 4 | 23.8 |  |  |
| 5 | 16.7 |  |  |
| 6 | 22.9 |  |  |
| 7 | 14.6 |  |  |

**Table S 6:** HI titres (log 2) of chickens in Chaling farm vaccinated through Ocular (tablet) method

| Chaling, Ocular | | | |
| --- | --- | --- | --- |
| HI titers (log 2) | % of chickens | % of 2^3 and above HI titer | % of 2^4 and above HI titer |
| 0 | 4.9 | 69 | 36 |
| 1 | 2.8 |  |  |
| 2 | 23.1 |  |  |
| 3 | 33.6 |  |  |
| 4 | 25.9 |  |  |
| 5 | 7.0 |  |  |
| 6 | 2.8 |  |  |
| 7 | 0 |  |  |

**Table S 7**: Field trial - vaccine efficacy data measured in HI at two testing sites.

|  | ***FIELD TRIAL SUMMARY*** | | | |
| --- | --- | --- | --- | --- |
|  |  | | | |
|  | **GOLDHUNGA** | | **CHALING** | |
|  | **Ocular, Tablet** | **Drinking Water, Lyophilized** | **Ocular, Tablet** | **Ocular, Lyophilized** |
| **HI titre (log 2)** | ***n*** | ***n*** | ***n*** | ***n*** |
| 0 | 0 | 3 | 7 | 1 |
| 1 | 7 | 12 | 4 | 1 |
| 2 | 20 | 15 | 33 | 17 |
| 3 | 26 | 22 | 48 | 35 |
| 4 | 56 | 59 | 37 | 42 |
| 5 | 40 | 68 | 10 | 16 |
| 6 | 52 | 49 | 4 | 9 |
| 7 | 35 | 12 | 0 | 0 |
| Blood in serum | 15 | 67 | 46 | 50 |
| No serum | 0 | 1 | 0 | 0 |

**Table S 8**: Post vaccination monitoring of chickens at Goldhunga farm- Morbidity and Mortality

| **Week** | **Biovac's Activities** | **Symptoms** | **Diagnosis** | **Intervention** | **Mortality** | **Cause of deaths** |
| --- | --- | --- | --- | --- | --- | --- |
| 1 | Swab collection for pre-screening (NDV & IAV) | Increased temperature, White discharge, ruffled feathers | Salmonellosis | None | 31 | Salmonellosis (white discharge), lack of proper ventilation |
| 2 | NDV I-2 Vaccination | White discharge, ruffled feathers | Salmonellosis | Cleaned water supply, better water management | 34 | Salmonellosis (white discharge) |
| 3 | Swab collection of NDV & IAV Screening | Stunted growth, wheezing, labored breathing | CRD | Cleaned water supply, better water management | 38 | Salmonellosis, CRD |
| 4 | Blood Collection for HI titre screening | Stunted growth, wheezing, labored breathing | CRD |  | 40 | Chronic respiratory disease (CRD) |
| 5 | Blood Collection for HI titre screening | Stunted growth, wheezing, labored breathing | CRD, overcrowding | Tylotar-D | 41 | CRD, overcrowding |
| 6 |  | Stunted growth, wheezing, labored breathing | CRD, overcrowding |  | 64 | CRD, overcrowding |
| 7 |  | Stunted growth, wheezing, labored breathing | CRD, overcrowding | Neodox | 93 | CRD, overcrowding |
| 8 |  | Stunted growth, wheezing, labored breathing | CRD, overcrowding |  | 121 | CRD, overcrowding |
| 9 |  | Stunted growth | CRD, overcrowding |  | 38 | CRD, overcrowding |

**Table S 9:** Post vaccination monitoring of chickens at Chaling farm- Morbidity and Mortality

| **Week** | **Biovac's Activities** | **Symptoms** | **Diagnosis** | **Intervention** | **Mortality** | **Cause of deaths** |
| --- | --- | --- | --- | --- | --- | --- |
| 1 | Swab collection for pre-screening (NDV & IAV) | Increased temperature, trampling | Overcrowding | None | 41 | Overcrowding, sudden drop in temperature |
| 2 | NDV I-2 Vaccination, | Sneezing, coughing | Possible reaction to vaccine | Space increased as chickens were still trampling over each other for food and water | 17 | Overcrowding |
| 3 | Swab collection of NDV & IAV Screening | Poor bird development, dilated abdomen, fatigue | Ascites (dilated abdomen due to fluid build up) | Liver tonic, Frusimide, & Colstron | 34 | Ascites |
| 4 | Blood Collection for HI titre screening | Poor bird development, dilated abdomen, fatigue | Ascites (dilated abdomen due to fluid build up) | Liver tonic, Frusimide, & Colstron | 25 | Ascites |
| 5 |  | Stunted growth, wheezing, labored breathing | CRD*, overcrowding | Tylotar -D | 24 | CRD |
| 6 |  | Stunted growth, wheezing, labored breathing | CRD, overcrowding | Tylotar -D | 43 | CRD |

**Table S 10**: NDV ELISA results- challenge trial (3 weeks)

| **SN** | **Sample ID** | **OD** | **Mean OD** | **ODPC/ODNC** | **SP ratio** | **Log Titre** | **Antibody Titre** | **Results** |  |
| --- | --- | --- | --- | --- | --- | --- | --- | --- | --- |
| 1 | NC | 0.044 | 0.0465 | 9.623655914 |  |  |  |  |  |
| 2 | NC | 0.049 |  |  |  |  |  |  |  |
| 3 | PC | 0.44 | 0.4475 |  |  |  |  |  |  |
| 4 | PC | 0.455 |  |  |  |  |  |  |  |
| 5 | 1 | 0.106 |  |  | 0.148379 | 2.691373 | 491.3292202 | Negative | VACCINATED |
| 6 | 2 | 0.598 |  |  | 1.375312 | 3.658401 | 4554.085125 | Positive |  |
| 7 | 3 | 0.232 |  |  | 0.462594 | 3.1852 | 1531.791098 | Positive |  |
| 8 | 4 | 0.27 |  |  | 0.557357 | 3.266133 | 1845.581188 | Positive |  |
| 9 | 5 | 0.283 |  |  | 0.589776 | 3.290687 | 1952.93043 | Positive |  |
| 10 | 6 | 0.389 |  |  | 0.854115 | 3.451516 | 2828.239629 | Positive |  |
| 11 | 7 | 0.573 |  |  | 1.312968 | 3.638254 | 4347.644276 | Positive |  |
| 12 | 8 | 0.58 |  |  | 1.330424 | 3.64399 | 4405.447713 | Positive |  |
| 13 | 9 | 0.363 |  |  | 0.789277 | 3.417229 | 2613.541146 | Positive |  |
| 14 | 10 | 0.237 |  |  | 0.475062 | 3.196751 | 1573.079268 | Positive |  |
| 15 | 11 | 0.243 |  |  | 0.490025 | 3.210218 | 1622.625072 | Positive |  |
| 16 | 12 | 0.107 |  |  | 0.150873 | 2.698611 | 499.5868541 | Negative |  |
| 17 | 13 | 0.169 |  |  | 0.305486 | 3.004992 | 1011.560159 | Positive |  |
| 18 | 14 | 0.3 |  |  | 0.63217 | 3.320834 | 2093.310207 | Positive |  |
| 19 | 15 | 0.057 |  |  | 0.026185 | 1.938045 | 86.7051565 | Negative | CONTTROL |
| 20 | 16 | 0.065 |  |  | 0.046135 | 2.184027 | 152.7662281 | Negative |  |
| 21 | 17 | 0.042 |  |  | -0.01122 | NA | NA | NA |  |
| 22 | 18 | 0.048 |  |  | 0.003741 | 1.092947 | 12.38645093 | Negative |  |
| 23 | 19 | 0.118 |  |  | 0.178304 | 2.771162 | 590.4208276 | Negative |  |
| 24 | 20 | 0.05 |  |  | 0.008728 | 1.460924 | 28.90171883 | Negative |  |
| 25 | 21 | 0.053 |  |  | 0.016209 | 1.729769 | 53.67462069 | Negative |  |
| 26 | 22 | 0.044 |  |  | -0.00623 | NA | NA | NA |  |
| 27 | 23 | 0.061 |  |  | 0.03616 | 2.078224 | 119.7356923 | Negative |  |
| 28 | 24 | 0.056 |  |  | 0.023691 | 1.894579 | 78.44752255 | Negative |  |
| 29 | 25 | 0.04 |  |  | -0.01621 | NA | NA | NA |  |
| 30 | 26 | 0.047 |  |  | 0.001247 | 0.615826 | 4.128816976 | Negative |  |
| 31 | 27 | 0.118 |  |  | 0.178304 | 2.771162 | 590.4208276 | Negative |  |

**Table S 11:** NDV ELISA results- challenge trial (4 weeks).

| **SN** | **Sample ID** | **OD** | **Mean OD** | **ODPC/ODNC** | **SP ratio** | **Log Titre** | **Antibody Titre** | **Results** |  |
| --- | --- | --- | --- | --- | --- | --- | --- | --- | --- |
| 1 | NC | 0.052 | 0.052 | 11.15384615 |  |  |  |  |  |
| 2 | NC | 0.052 |  |  |  |  |  |  |  |
| 3 | PC | 0.58 | 0.58 |  |  |  |  |  |  |
| 4 | PC | 0.58 |  |  |  |  |  |  |  |
| 5 | 1 | 0.612 |  |  | 1.410224 | 3.669288 | 4669.692 | Positive | Vaccinated |
| 6 | 2 | 1.165 |  |  | 2.789277 | 3.965492 | 9236.164 | Positive |  |
| 7 | 3 | 0.699 |  |  | 1.627182 | 3.731436 | 5388.106 | Positive |  |
| 8 | 4 | 0.896 |  |  | 2.118454 | 3.846019 | 7014.86 | Positive |  |
| 9 | 5 | 0.713 |  |  | 1.662095 | 3.740656 | 5503.713 | Positive |  |
| 10 | 6 | 0.881 |  |  | 2.081047 | 3.838282 | 6890.996 | Positive |  |
| 11 | 7 | 0.997 |  |  | 2.370324 | 3.894808 | 7848.881 | Positive |  |
| 12 | 8 | 0.942 |  |  | 2.233167 | 3.868921 | 7394.711 | Positive |  |
| 13 | 9 | 0.998 |  |  | 2.372818 | 3.895264 | 7857.139 | Positive |  |
| 14 | 10 | 0.647 |  |  | 1.497506 | 3.695369 | 4958.709 | Positive |  |
| 15 | 11 | 0.606 |  |  | 1.395262 | 3.664656 | 4620.146 | Positive |  |
| 16 | 12 | 0.36 |  |  | 0.781796 | 3.413093 | 2588.768 | Positive |  |
| 17 | 13 | 0.364 |  |  | 0.791771 | 3.418599 | 2621.799 | Positive |  |
| 18 | 14 | 0.68 |  |  | 1.5798 | 3.718602 | 5231.211 | Positive |  |
| 19 | 15 | 0.059 |  |  | 0.031172 | 2.013766 | 103.2204 | Negative | Control |
| 20 | 16 | 0.056 |  |  | 0.023691 | 1.894579 | 78.44752 | Negative |  |
| 21 | 17 | 0.053 |  |  | 0.016209 | 1.729769 | 53.67462 | Negative |  |
| 22 | 18 | 0.057 |  |  | 0.026185 | 1.938045 | 86.70516 | Negative |  |
| 23 | 19 | 0.046 |  |  | -0.00125 | NA | NA | NA |  |
| 24 | 20 | 0.065 |  |  | 0.046135 | 2.184027 | 152.7662 | Negative |  |
| 25 | 21 | 0.058 |  |  | 0.028678 | 1.977553 | 94.96279 | Negative |  |
| 26 | 22 | 0.05 |  |  | 0.008728 | 1.460924 | 28.90172 | Negative |  |
| 27 | 23 | 0.059 |  |  | 0.031172 | 2.013766 | 103.2204 | Negative |  |
| 28 | 24 | 0.056 |  |  | 0.023691 | 1.894579 | 78.44752 | Negative |  |
| 29 | 25 | 0.049 |  |  | 0.006234 | 1.314796 | 20.64408 | Negative |  |
| 30 | 26 | 0.115 |  |  | 0.170823 | 2.752546 | 565.6479 | Negative |  |
| 31 | 27 | 0.058 |  |  | 0.028678 | 1.977553 | 94.96279 | Negative |  |
| 31 | 31 | 0.052 |  |  | 0.013716 | 1.657218 | 45.41699 | Negative | Hatched Chicken |
| 32 | 32 | 0.048 |  |  | 0.003741 | 1.092947 | 12.38645 | Negative |  |
| 33 | 33 | 0.049 |  |  | 0.006234 | 1.314796 | 20.64408 | Negative |  |
| 34 | 34 | 0.047 |  |  | 0.001247 | 0.615826 | 4.128817 | Negative |  |
| 35 | 35 | 0.05 |  |  | 0.008728 | 1.460924 | 28.90172 | Negative |  |
