# Supplementary material for: Newcastle disease burden in Nepal and efficacy of Tablet I2 vaccine in commercial and backyard poultry production
Source: PLoS One. 2023 Mar 10;18(3):e0280688. doi: 10.1371/journal.pone.0280688 (PMC10004539; doi:10.1371/journal.pone.0280688)
Supplement: S2 File — (DOCX) [file pone.0280688.s002.docx]

**Supplementary Data (Figures)**


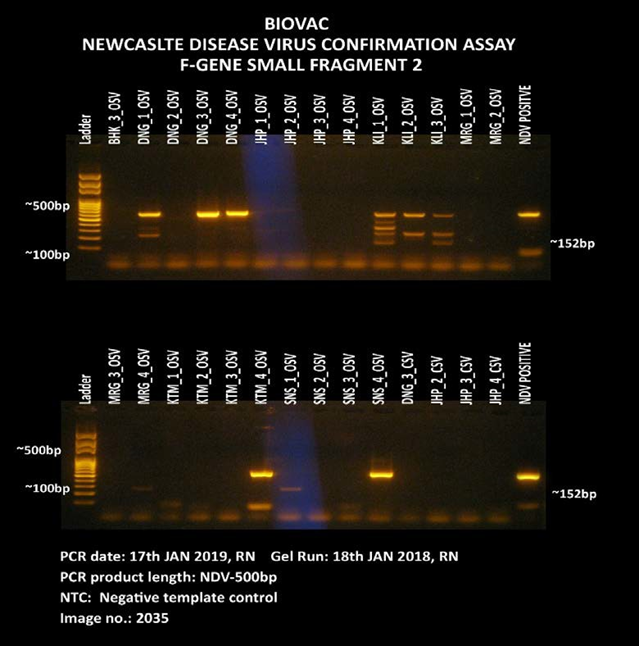


**Figure S 1**: PCR products (1.5% agarose Gel)- 500 bp F gene fragment 2 positive samples. Each district coded with three letter code had four farms numbered 1 to 4. Each sample represents a farm pooled in a single tube and screened. (The gel was run with ladder on first well and Negative control followed by positive controls on last well (from left to right). [Dang (DNG_1, DNG_3, DNG_4), Kathmandu (KTM_4) and Sunsari (SNS4) District respectively]


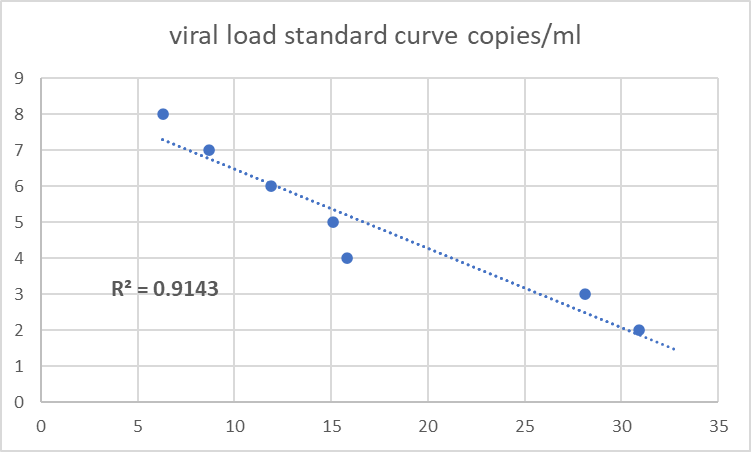

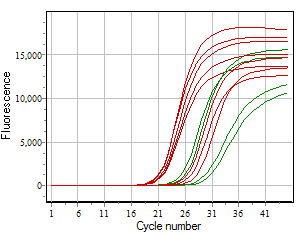

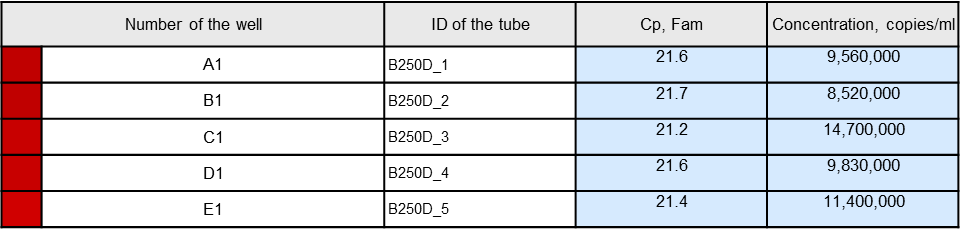


**Figure S 2** Quantitative real time PCR for quantitation of NDV (viral load) in each vaccine dose. The RNA standards for viral load determination were prepared by using Newcastle disease virus (Path-NDV-standard-Genesig, UK) quantitative standards as a reference. The RNA for standards were prepared by performing 10-fold dilutions from sample to have known viral copies of 10^8^/ml. A total of 8 dilutions of the RNA samples were prepared followed by cDNA preparation using iScript cDNA synthesis kit. The quantitative PCR was performed by using SYBR™ Green PCR Master Mix (Catalog number: 4309155-ThermoFishcer, USA) at PCR conditions: initial denaturation at 95̊C for 7 minutes, denaturation at 95̊C for 10 seconds, annealing at 58̊C for 15 seconds and extension at 72̊C for 20 seconds followed by final extension at 72̊C for 5 minutes. The PCR condition was performed for 40 cycles. The standard curve was prepared using the Ct values and the corresponding known dilution of viral copies; the coefficient of determination was 0.9143. The viral load of five Ranigoldhunga^TM^ vaccine samples were determined using the same standard curve as reference following the above mentioned SYBR Green PCR protocol. The result showed the Ct values of the five samples ranged from 21.2 to 21.7- with viral load ranging from 8.5 million to 11.4 million copies of virus per ml of the sample.
