# Supplementary material for: Newcastle disease burden in Nepal and efficacy of Tablet I2 vaccine in commercial and backyard poultry production
Source: PLoS One. 2023 Mar 10;18(3):e0280688. doi: 10.1371/journal.pone.0280688 (PMC10004539; doi:10.1371/journal.pone.0280688)

Figure 7 a) Top Image Commercial Farm PCR result on elctrophoresis Raw Image

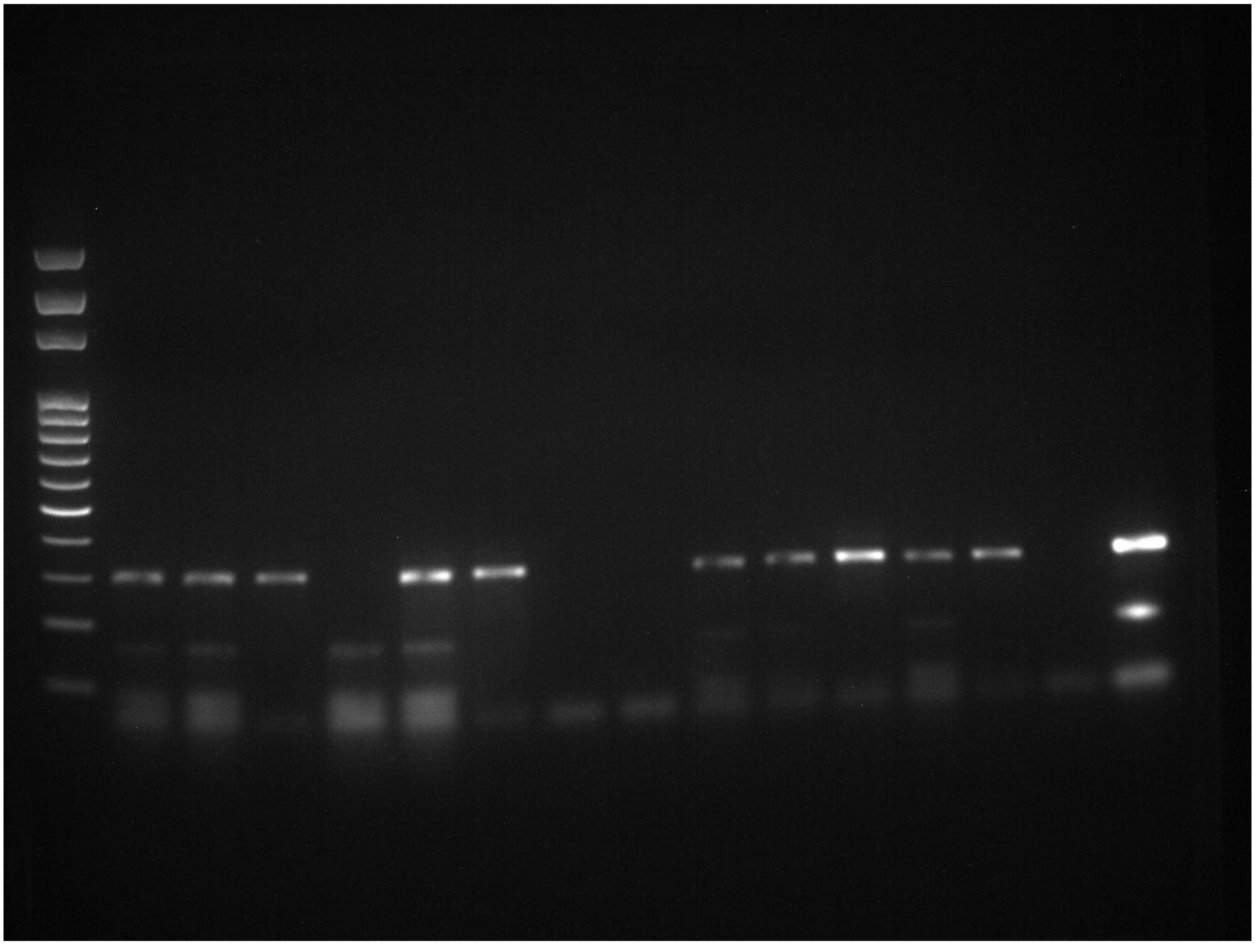

Figure 7 b) Middle Image Commercial Farm PCR result on elctrophoresis Raw iMAGE

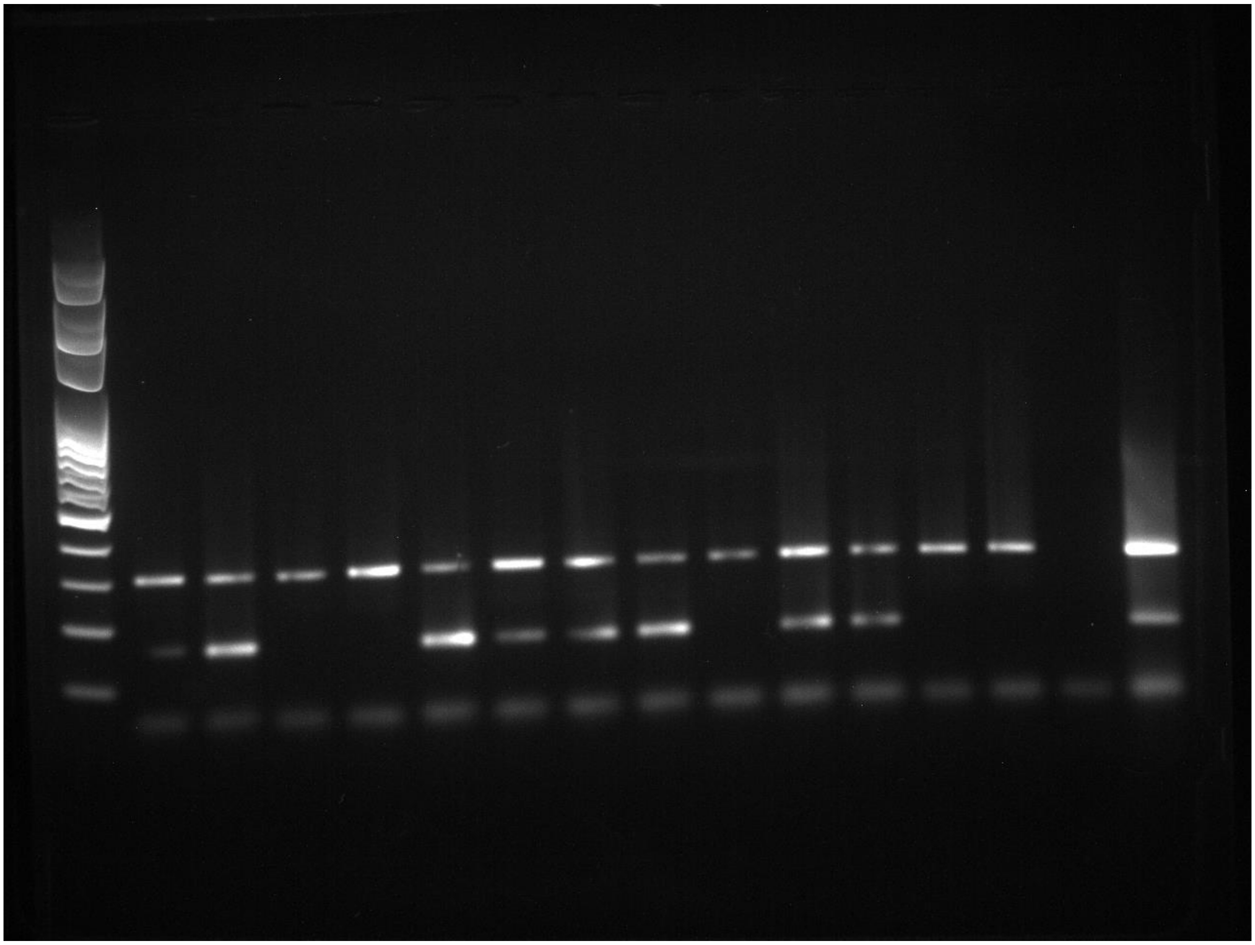

Figure 7 c) Bottom Image Commercial Farm PCR result on elctrophoresis Raw iMAGE

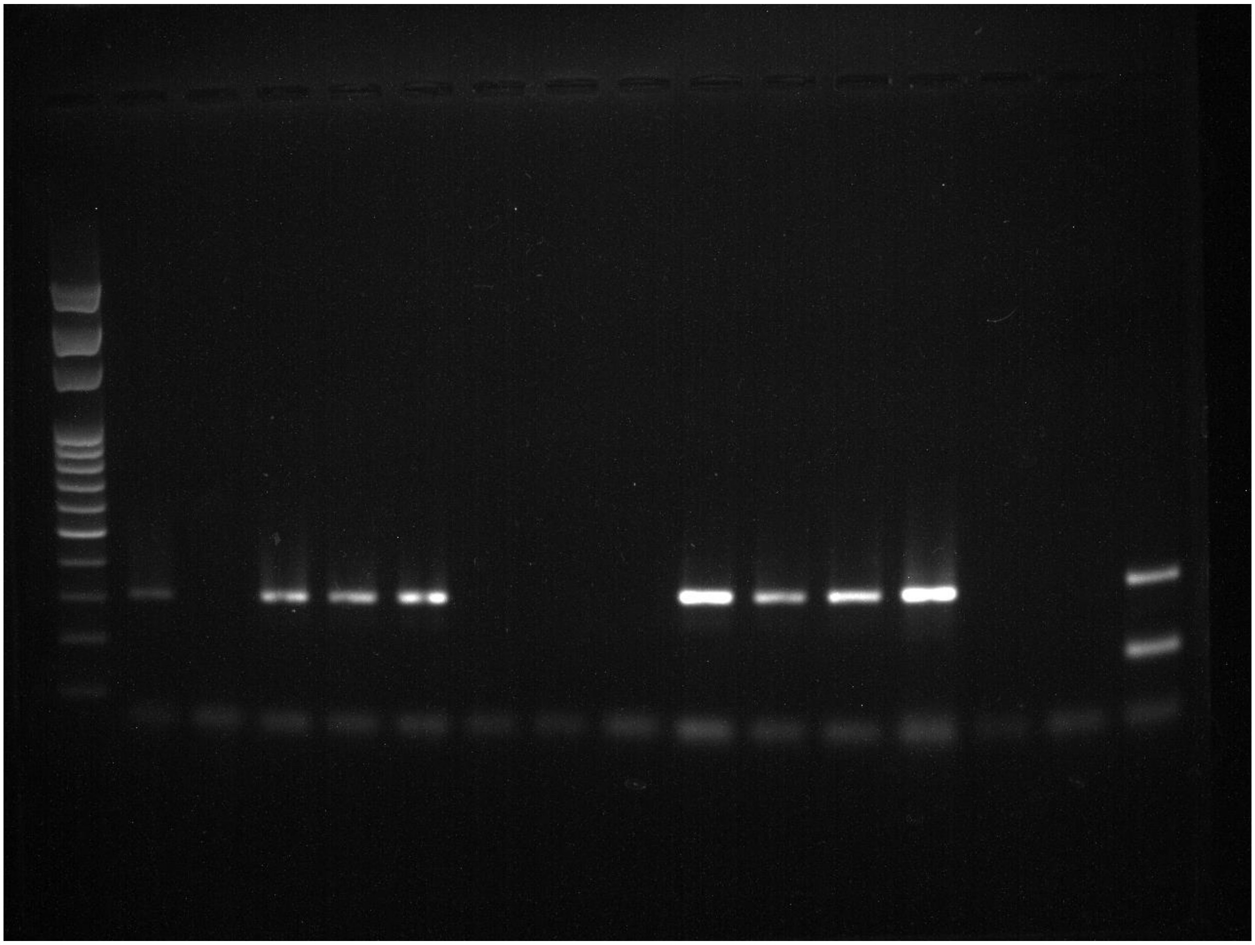

Figure 8 a) Top Image Backyard Farm PCR result on elctrophoresis Raw iMAGE

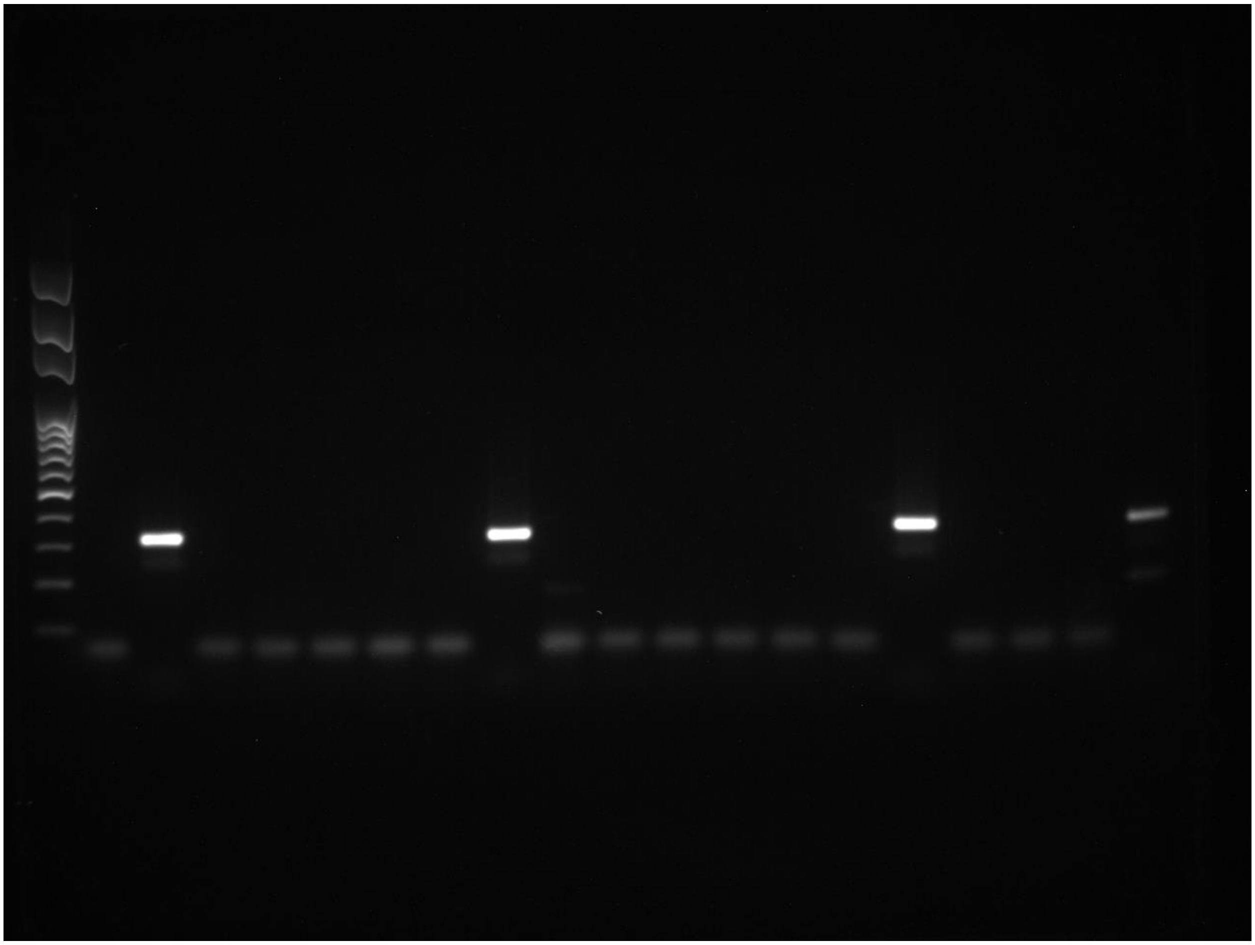

Figure 8 b) Bottom Image Backyard Farm PCR result on elctrophoresis Raw iMAGE

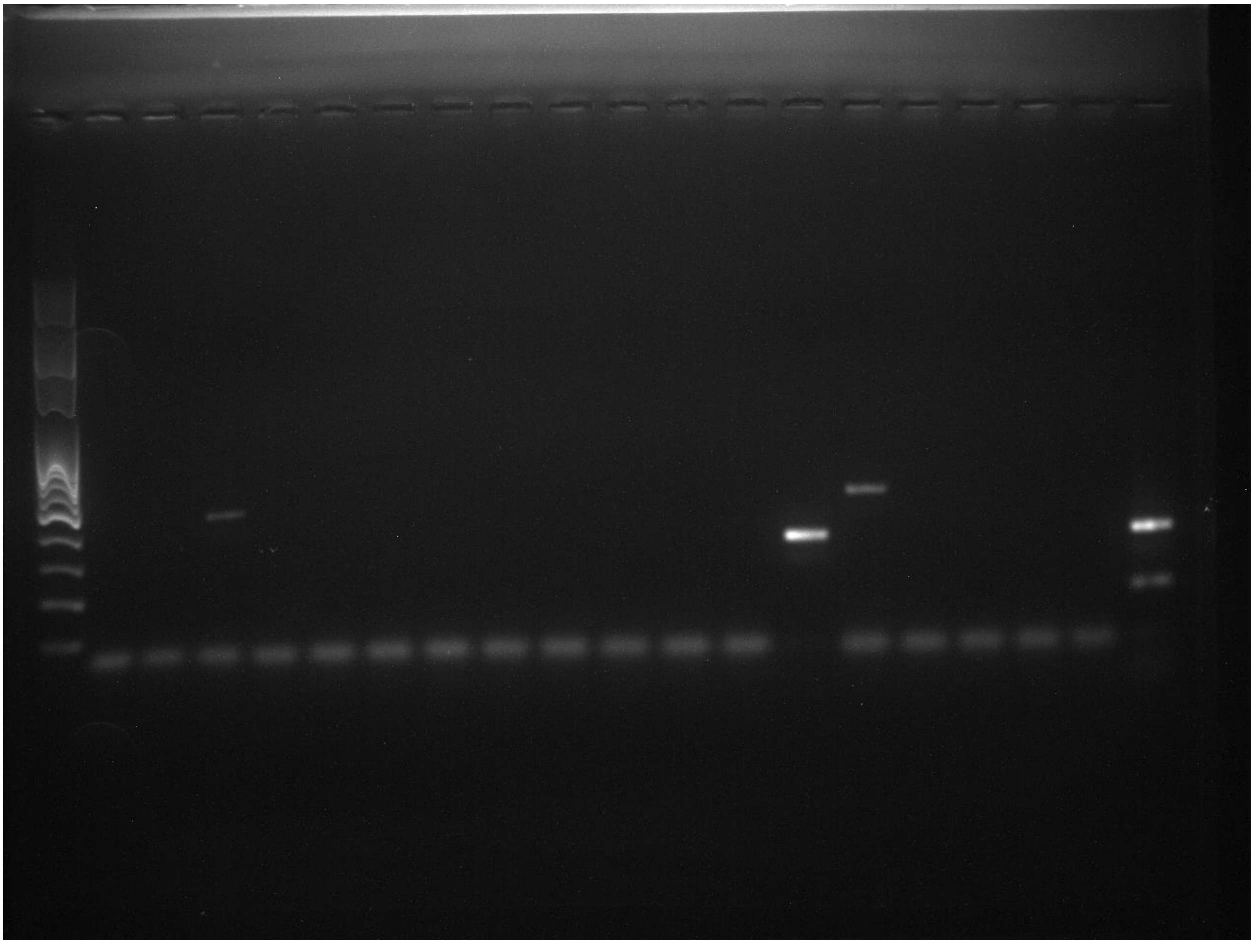

Supplement: S3 File — (PDF) [file pone.0280688.s003.pdf]
