# Supplementary material for: Newcastle disease burden in Nepal and efficacy of Tablet I2 vaccine in commercial and backyard poultry production
Source: PLoS One. 2023 Mar 10;18(3):e0280688. doi: 10.1371/journal.pone.0280688 (PMC10004539; doi:10.1371/journal.pone.0280688)
Supplement: Attachment — Submitted filename: 82303.pdf [file pone.0280688.s004.pdf]

# Field Verification Trial of ND I-2 Vaccine in Nepal

M. P. Acharya[1*], S. K. Adhikari[2], H. Awasthi[2], A. Jha[3]and U. M. Singh[4]

1. Animal Health Research Division, NARC, Nepal.
2. Department of Livestock Service.
3. Regional Agricultural Research Station, Parwanipur.
4. Himalayan College of Agricultural Science and Technology. Kalanki.

*Corresponding author: mpacharya555@yahoo.com; mpacharya@narc.gov.np

## ABSTRACT

*A study was conducted to assess the overall performance of ND I-2 vaccine in Nepalese context to prevent Newcastle disease (ND) particularly in backyard chicken. The experimental studies were conducted at Animal Health Research Division (AHRD), NARC and field verification were carried out in different eco-zones of the country. Experiments were divided in three segments: evaluation of thermostability, longevity and field verification trial in backyard chickens. The ND I-2 vaccine was prepared at Central Biological Production Laboratory (CBPL), Tripureshwor. In thermostability evaluation antibody titer and mortality percentage showed that ND I-2 vaccine exposed up to 30$^0$C for seven days was found effective in prevention of Newcastle Disease in village chickens. Evaluation of longevity of immunity showed that till 90 days there was protective immunity against ND and in field trials when vaccine delivered without maintaining cold chain, the vaccine gave protective immunity in mid hills and high hills region but not enough immunity in the terai region where the temperature was above 30$^0$ Celsius. This study clearly indicated that thermostable ND I-2 vaccine can be used without cold chain in high hills and mid hills in all seasons and only in winter season in the terai region of Nepal (temp. less than 30$^0$C). In summer season, vaccination in the terai region could not give protective immunity (temp. more than 30$^0$C) without maintaining cold chain.*

**Keywords:** $EID_{50}$, HI, Specific immunity, Thermostable ND I-2 vaccine

## INTRODUCTION

Nepal is an agricultural based country, agriculture contribute 28% in national GDP. Livestock shares 12% and poultry sector alone contributes 4% in GDP (ABPSD, 2016). In total population of poultry, backyard chickens comprise 46% and commercial chickens comprise 56% (Parajuli 2008). Newcastle disease (ND) has great economic significance to the poultry industry worldwide. ND is a highly contagious and fatal viral disease that affects birds of all age groups of most species. The disease is frequently responsible for devastating losses in poultry (Alexander *et.al., 2004*). The causative agent of ND in birds is an avian paramyxovirus serotype 1 (APMV-1), which is the member of the genus Avulavirus of the Paramyxoviridae family (Senne *et al*., 2004). Depending upon the pathotype involved and susceptibility of the flock, the virus causes mortality ranging up to 100%. For this reason, it is included in list A of the Office International des Epizooties (OIE, 2002).

Vaccination is the most cost-effective method for controlling ND and has been used successfully throughout the world (Alexander *et.al.,* 1997). There are two types of vaccines: thermostable vaccine and thermolabile vaccine (FAO, 2002). The ND thermotolerant vaccines are among the attenuated vaccines that offer an appropriate technology for ND control in rural free-range poultry management system where cold chain facilities, logistics management and husbandry factors need consideration (Alder and Spradbrow, 2001). Thermostable ND vaccine exhibit a relative resistance to inactivation and exposure to elevated temperature. Strain of ND virus vary in thermostability. Thermostable vaccines are prepared from a strain of Newcastle disease virus that retains the ability to infect cells after storage outside the cold chain for a short period of times.

In our context, particularly for rural chickens which comprises more than 46% poultry population, thermostable vaccine is more suitable than thermolabile vaccine due to various reasons like poor electrification infrastructures, frequent power cut-offs, scattered and small-scale rearing system and lack of technicians for injectable vaccines. Besides these reasons, thermostable vaccine can be used irrespective of chicken age and longevity of protective immunity, at least three month in Nepalese context (Nyaupane *et.al.,* 2016). Whereas heat labile (thermolabile) vaccines which are being used in commercial chickens right now needs cold chain system. During frequent load-shedding and even without refrigeration facility, heat labile vaccine fails to give protective immunity against ND to chickens. Therefore, thermostable ND vaccine is very useful in Nepal. There are not enough research reports on control of ND in village chicken in Nepal. However, the evaluation of thermostability and longevity of ND, I-2 vaccine conducted at Animal Health Research Division, NARC has showed the very promising result and recommended for field verification (Acharya *et.al.,* 2016).

In this study, field verification trials of ND, I-2 vaccine produced at CBPL, Tripureshwor were performed in backyard chicken in three eco-zones of the country including five districts namely Bara, Sunsari, Kavre, Jajarkot and Jumla. In Jumla and Jajarkot, chicks distributed by Agriculture and Food Security Project (AFSP) were used for sampling, vaccination and sero-monitoring whereas chickens maintained by farmers were used in other locations irrespective of age.

## METHODOLOGY

### Site of Trials

The field trials were carried out at five locations representing five districts covering three agro-ecological zones. Two districts each from Terai (Madhesa village, Sunasari and Fattepur village, Bara) and mid hills (Tinpiple village, Kavre and Pokhara, Jajarkot) and one district from high hills (Bohara village, Jumla). In Jumla and Jajarkot field trials were conducted on those chicks which were distributed by Agriculture and Food Security Project (AFSP). Trial was conducted in summer season when the temperature of Terai region was above $30^0$C.

## Experimental Design

Ten households having at least 15 chickens were selected randomly for sampling purpose however, the vaccination was carried out in all birds of village irrespective of the age. Vaccine having approximately $10^6$ $EID_{50}$ /0.1ml were delivered by intra-ocular route at the dose rate of 100 µl/bird. Blood samples were collected at day-one before vaccination (1 DprV) and 30 days-post vaccination (30 DPV). Pre-vaccination and post vaccination sampling were carried out in farmer's field for monitoring antibody to ND by haemagglutination inhibition (HI) test. Results were noted and analyzed for efficacy of ND I-2 vaccine in village chickens in field condition ($EID_{50}$ calculation, Reed *et al.*, 1938).

**Protocol for Field Trial** (ACIAR, 2002)

i. 30 blood samples were collected from each trial site before vaccination for base line antibody evaluation
ii. 20 households having minimum of 10-15 chicken irrespective of age of chicken (at least 450-500 chicken) were selected for vaccination
iii. Vaccine reconstituted in given diluents having approximately $10^6$ $EID_{50}$ / 0.1ml which has already calculated while titration of vaccine.
iv. Vaccine was delivered @ the dose rate of 100 µl / bird intra-ocular using micropipette.
v. Blood samples were collected 30 days post immunization (30 DPI).
vi. Sixty blood samples were collected from adult birds of each sites.

## Haemagglutination Inhibition (HI) Assay

Haemagglutination (HA) and HI tests were conducted according to OIE Manual, 2012. All the collected sera were stored in - $40^0$C until use. Before HI assay, sera were inactivated at $56^0$C for 30 minutes. Sera collected before vaccination were analyzed for baseline antibody titer monitoring. The antibody titer in sera was determined by using 4 HA unit with two-fold serial dilution as recommended by the World Organization for Animal Health (OIE, 2012). Published cut off value was used for the protective HI antibody titer (HI titer$\geq$ log$2^3$) (Spradbrow *et al.,* 1993; VanBoven *et al.,* 2008).

## RESULTS

## Immunization with ND I-2 Vaccines in Birds of Field Verification Site

ND I-2 vaccine delivery, sampling and antibody titers calculation were performed as described in methodology along with experimental protocols. The comparative antibody titers of field birds of different locations are shown in Tables 1 to 5.

Table 1: Antibody titer and protective level of specific immunity to NDV in birds at Tinpiple, Kavre district.

| Days of Vaccination | Antibody titer using HI test | | | | | | | | | Protective Level of specific immunity of NDV |
|---|---|---|---|---|---|---|---|---|---|---|
| | 1:0 | 1:4 | 1:8 | 1:16 | 1:32 | 1:64 | 1:128 | 1:256 | ≥1:256 | |
| Pre-vac (1 DPrV) | 6 | 10 | 11 | 3 | - | - | - | - | - | 10 % |
| Postvac (30 DPV) | - | - | - | - | 4 | 36 | 14 | 6 | - | 100 % |

DPrV: Days Pre-Vaccination. DPV: Days Post Vaccination.

Table 2: Antibody titer and protective level of specific immunity to NDV in birds at Fattepur, Bara district.

| Days of Vaccination | Antibody titer using HI test | | | | | | | | | Protective Level of specific immunity of NDV |
|---|---|---|---|---|---|---|---|---|---|---|
| | 1:0 | 1:4 | 1:8 | 1:16 | 1:32 | 1:64 | 1:128 | 1:256 | ≥1:256 | |
| Pre-vac (1 DPrV) | 16 | 10 | 4 | - | - | -- | - | - | - | 0 % |
| Postvac (30 DPV) | - | 16 | 14 | 4 | 22 | - | 4 | - | - | 50 % |

DPrV: Days Pre-Vaccination. DPV: Days Post Vaccination.

Table 3: Antibody titer and protective level of specific immunity to NDV in birds at Madhesa, Sunasari district.

| Days of Vaccination | Antibody titer using HI test | | | | | | | | | Protective Level of specific immunity of NDV |
|---|---|---|---|---|---|---|---|---|---|---|
| | 1:0 | 1:4 | 1:8 | 1:16 | 1:32 | 1:64 | 1:128 | 1:256 | ≥1:256 | |
| Pre-vac (1 DPrV) | 20 | - | 8 | 2 | - | -- | - | - | - | 6.67 % |
| Postvac (30 DPV) | - | - | 24 | - | 8 | 6 | 22 | - | - | 60 % |

Table 4; Antibody titer and protective level of specific immunity to NDV in birds at Bohara Village, Jumla district.

| Days of Vaccination | Antibody titer using HI test | | | | | | | | | Protective Level of specific immunity of NDV |
|---|---|---|---|---|---|---|---|---|---|---|
| | 1:0 | 1:4 | 1:8 | 1:16 | 1:32 | 1:64 | 1:128 | 1:256 | ≥1:256 | |
| Pre-vac (1 DPrV) | 8 | 10 | 9 | 3 | - | -- | - | - | - | 10 % |
| Postvac (30 DPV) | - | - | 8 | 6 | 6 | 6 | 4 | 30 | - | 86.66 % |

Table 5: Antibody titer and protective level of specific immunity to NDV in birds at Pokhara, Jajarkot district.

| Days of Vaccination | Antibody titer using HI test | | | | | | | | | Protective Level of specific immunity of NDV |
|---|---|---|---|---|---|---|---|---|---|---|
| | 1:0 | 1:4 | 1:8 | 1:16 | 1:32 | 1:64 | 1:128 | 1:256 | ≥1:256 | |
| Pre-vac (1 DPrV) | 10 | 12 | 8 | - | - | -- | - | - | - | 0 % |
| Postvac (30 DPV) | - | 4 | 5 | 6 | 4 | 8 | 6 | 10 | 17 | 85 % |

## DISCUSSION

Village chickens are major component of poultry production in Nepal. However, there is always threat of infectious poultry diseases among which Newcastle disease (ND) is responsible for large economic loses and vaccination is only the effective approach to control.

In this study, birds vaccinated using thermostable ND, I-2 vaccine have shown different protection responses in different locations ranging from 50 to 100% protection (Table 1 to 5). The protective immunity in Kavre district was highest (100%) followed by Jumla (86.6%); Jajarkot (85%); Sunsari (60%); and Bara (50%) respectively. In few birds, there was certain level of immunity detected before vaccination which might be due to previous exposure to ND. This result is similar to other reports which discussed protective immunity at various level (up to 100%) (Acharya *et al.*, 2016; Nyaupane *et. al.*, 2016).

In previous study, when evaluating thermostability of ND I-2 vaccine, kept in room temperature (25-30$^0$C) for 7 days had protected ≥80% experimental chicken against challenged field isolate of ND. Likewise, vaccine kept in incubator (37$^0$C) for 7 days could not protect enough against field virus challenge (Acharya *et al.*, 2016). This fact is very much similar to present findings where HI test results have shown 100% protective immunity in Kavre, 86.6% in Jajarkot, 85% in Jumla, 50% in Bara and 60% in Sunsari. In both Terai region the temperature was above 35$^0$C and in mid hill and high hill region (viz. Kavre, Jajarkot and Jumla) temperature was below 30$^0$C range.

In another study about longevity of protective immunity, this vaccine produced specific immunity against ND for at least 90 days (3 month) after vaccination in village chicken (Nyaupane et al, 2016). Whereas another study has claimed that protective immunity lasts for four months and hence it is enough to repeat vaccine in every 4 months (Alders and Spradbrow, 2001). Protective immunity is generally accepted as corresponding to a hemagglutination inhibition titer above 8 ($log_2{}^3$) (Bell *et. al.*, 1991). In this results protective HI titer varied in mid hill and hilly areas also, like 100 % immunity seen in Kavre unlike Jumla and Jajarkot. The reasons behind this variation in immune response and longevity of immunity could be due to the immune status and health status of the bird at the time of exposure to the immune stimulus, the nature of the immune stimulus and slight variation in travelling days from research center to trial sites. Generally, in vaccinal immunity if more than 80% protective immunity is achieved in experimental birds, it is considered as effective vaccine to control the disease. Therefore, in this study, vaccine has shown effective response in three districts - Kavre, Jumla and Jajarkot and not protective enough in Bara and Sunsari districts during summer.

Village chickens are not frequently vaccinated against common diseases. Major constraint noticed is the lack of vaccine suited to our village condition. Vaccines which are currently in use for control of ND are targeted to commercial poultry. Manufacturer are providing heat labile ND vaccines in multi-dose vials such as 1000 dose and 2500 doses and need to maintain within cold chain ($4^0$C to $8^0$C) from manufacture to administration to the chickens which is not easy to maintain cold chain at present condition due to poor electrification network. In contrast, village chickens reared in small scale in scattered form and in free-range system, for these chicken available vaccines are inappropriate. Therefore, to address these various constraints, thermostable ND I-2 vaccine has been used and demonstrated satisfactory protection against velogenic NDV in many Asian and African countries (Vietnam, Malaysia, Tanzania, Malawi, Mozambique, Myanmar, and South Africa). From the studies conducted in Nepal, ND I-2 vaccine seemed to be avirulent, immunogenic and highly protective against virulent isolates of NDV (Shrestha *et. al.*, 2008; Acharya *et. al.*, 2016; Nyuapane *et. al.*, 2016) and hence it may be a suitable vaccine to use in village chickens to protect them against ND.

## CONCLUSION

Field trial result showed that ND, I-2 vaccine was effective for village chicken irrespective of age and no necessity of maintenance of cold chain for 7 days during transportation and administration of vaccine where the temperature ranges below $30^0$C. However, it is recommended that earlier vaccination yields better result, which will certainly assure high vaccinal response. In case of terai region where temperature goes above $30^0$C, vaccination can be conducted in winter only. This vaccine is potent and adoptable. This vaccine does not give lifelong protection and must be repeated in every three months in our context. Adaptation of this vaccine can be a milestone towards preventing ND in village chicken which will improve the livelihood of rural communities and empower the women farmers along with high value protein supplementation.

## REFERENCES

Acharya M.P; Adhikari S.K, Singh U.M, Gautam S.P., and S Shrestha (2016). Evaluation of Thermostability of ND, I-2 Vaccine for Newcastle Disease Prevention in Village Chicken. *Nepalese Vet...J.* 33; 96-103.

ACIAR. (2002). Control of Newcastle disease in village chickens. A Laboratory Manual. Canberra, Australian Centre for International Agricultural Research. ACIAR Monograph No. 87 142pp.

Agri-Business Promotion and Statistics Division (2016). Ministry of Agriculture and Livestock Development

Alders, R.G. and Spradbrow, P.B. (2001). *Controlling Newcastle Disease in Village Chickens:*a field manual. Canberra, Australian Centre for International Agricultural Research. Monograph82. 112pp.

Alexander, D. J. (1997). Newcastle disease and other paramyxovirus Infections. *In*: B. W. Calnek, H. J. Barnes, C. W. Beard, L. R. McDougal, and Y. M. Saif. (Ed.) *Diseases of Poultry*, ($10^{th}$ Ed.) Iowa State University Press, USA, IA: pp 541-569

Alexander, D. J., Bell, J. G. and Alders, R. G. (2004). *A technology review*. Newcastle disease with special emphasis on its effect on village chicken (No 161). Food and Agri-Org.

Bell, J. G., Nicholas, P. J., Norman, C., Cooper, K. and Cross, G. M. (1991a). The serological responses of chickens to mass vaccination with a live V4 Newcastle virus vaccine in the field and in the laboratory. 1. Meat chickens. *Australian Veterinary Journal* 68: 85-89.

FAO. (2002). A basic laboratory manual for the small-scale production and testing of I-2 Newcastle Disease Vaccine. RAP Publication (2002/22); Food and Agriculture Organization, Rome, Italy. ISBN 974-7946- 26-2. 8p.

Nyaupane,S., Pokhrel, B. B. and Acharya, M. P. (2016). A Study to Estimate Longivity of Thermostable Newcastle Disease Vaccine (Strain I-2) in Village Chicken of Nepal. *Advance in Life Sciences* 2016, 6 (2): 45-48.

OIE (2012). Terrestrial Animal Health Manual Chapter 3.3.14-Newcastle Disease. (Infection with Newcastle Disease Virus).

Parajuli, D.P. (2008). Small scale rural poultry production and its contribution to poverty alleviation. In *Proceeding of $8^{th}$ National Conference of NVA.* pp 20.

Reed, L. J. and Muench, H. (1938). A simple method for estimating fifty percent end point. *AM, J, Hyg*, 27,493-497.

Senne, D.A., King, D.J. and Kapczynski, D.R. (2004). Control of Newcastle disease by vaccination. *Developments in Biological (Basel),* 119,165-170.

Shrestha, S., Parajuli, B., Gautam, S., Bhail, R. and Maharjan, K. K. (2008). Development of ND, I-2 vaccine for control of ND in village chicken in Nepal. *Proceeding of $8^{th}$ National Veterinary Conference,* 8-10 May, 2008: 60-64.

Spradbrow, P.B. (1993). Newcastle disease in village chickens. Poultry Sci. Review 5: 57-96.

Van Boven, M., Bouma, A., Fabri, T. H., Katsma, E., Hartog, L. and Koch, G. (2008). Herd Immunity to Newcastle disease virus in poultry by vaccination. *Avian Pathol*; 37; 1-5.
