# Supplementary material for: Newcastle disease burden in Nepal and efficacy of Tablet I2 vaccine in commercial and backyard poultry production
Source: PLoS One. 2023 Mar 10;18(3):e0280688. doi: 10.1371/journal.pone.0280688 (PMC10004539; doi:10.1371/journal.pone.0280688)
Supplement: Attachment — Submitted filename: 10.1.1.469.3015.pdf [file pone.0280688.s005.pdf]

Veterinary Research Communications, 24 (2000) 275–286
© 2000 Kluwer Academic Publishers. Printed in the Netherlands

Pathotyping of Newcastle Disease Viruses by RT-PCR and Restriction Enzyme Analysis

T. Nanthakumar, R.S. Kataria, A.K. Tiwari*, G. Butchaiah and J.M. Kataria
National Biotechnology Centre, Indian Veterinary Research Institute, Izatnagar U.P. 243 122, India
**Correspondence: E-mail: ivri@x400.nicgw.nic.in*

Nanthakumar, T., Kataria, R.S., Tiwari, A.K., Butchaiah, G. and Kataria, J.M., 2000. Pathotyping of Newcastle disease viruses by RT-PCR and restriction enzyme analysis. *Veterinary Research Communications*, **24(3)**, 275–286

ABSTRACT

The technique of RT-PCR and restriction enzyme analysis was standardized to detect and differentiate Newcastle disease viruses. Digestion of RT-PCR-amplified, F gene sequences encoding for the cleavage activation sites of fusion protein with restriction enzymes *Alu*I, *Bgl*I, *Hae*III, *Hin*fI, *Hha*I, *Rsa*I, *Sty*I and *Taq*I was carried out in order to characterize Newcastle disease viruses of varying pathogenicity. Restriction enzyme digestion of the amplicons by *Bgl*I and *Hha*I could group eight viruses, both field isolates and known vaccine strains, into lentogenic, mesogenic and velogenic pathotypes. By employing this technique directly on a clinical sample, Newcastle disease virus of the lentogenic pathotype could be detected.

Keywords: amplicons, classification, diagnosis, Newcastle disease, pathotype, polymerase chain reaction, restriction enzymes

Abbreviations: dNTPs, deoxynucleoside triphosphate; DTT, dithiothreitol; ECE, embryonated chicken eggs; ICPI, intracerebral pathogenicity index; IVPI, intravenous pathogenicity index; MDT, mean death time; MMLV, Moloney murine leukaemia virus; ND, Newcastle disease; NDV, Newcastle disease virus; RT, reverse transcription; PCR, polymerase chain reaction; SPF, specific pathogen-free

INTRODUCTION

Newcastle disease (ND) is a highly contagious viral disease of poultry affecting chickens, pigeons, turkeys, guinea fowl, Japanese quail and many wild birds of all ages, worldwide (Alexander, 1991). The disease is characterized by respiratory distress, depression, diarrhoea and impairment of the central nervous system. The mortality in an affected flock may be up to 90–100%, resulting in heavy economic losses. Newcastle disease virus (NDV), the causative agent of the disease, is classified in the genus *Rubulavirus* of the family *Paramyxoviridae* and is an enveloped virus, having a non-segmented, 15 kb single-stranded RNA genome of negative polarity (Mayo and Pringle, 1998; Alexander, 1990). The genome of NDV encodes for six major structural and non-structural proteins. The fusion protein, along with haemagglutinin-neuraminidase, serves as the target for the immune response of the host. Fusion protein, which initiates infection by fusion with the host cell membrane, is synthesized as a F_0

precursor, which is activated only after cleavage into F_1 and F_2 fragments by host cell proteases (Nagai *et al.*, 1976; Umino *et al.*, 1990).

Based on their pathogenicity, ND viruses have been categorized into lentogenic, mesogenic and velogenic pathotypes, which also differ in the number of basic amino acids at the cleavage site and/or by amino acid substitution at position 124 in their fusion protein (Toyoda *et al.*, 1987; Collins *et al.*, 1993; Seal *et al.*, 1995). Owing to the presence of many pathotypes and the almost universal use of live NDV vaccines, the mere isolation and identification of virus are inadequate for disease control and further characterization is required. Detection and differentiation of NDV pathotypes are conventionally based on isolation of the virus in embryonated chicken eggs, followed by *in vivo* tests, such as the intracerebral pathogenicity index (ICPI), the intravenous pathogenicity index (IVPI) and the mean death time (MDT) in SPF birds/embryos. These tests are labour intensive, time consuming, expensive and inhumane (Alexander and Allan, 1974; Seal *et al.*, 1995). To differentiate pathotypes of NDV, many recent workers have used the relatively simple methods of reverse transcription–polymerase chain reaction (RT-PCR) and restriction enzyme digestion of different gene sequences, mainly the F gene encoding the cleavage site of fusion protein (Seal *et al.*, 1995; Marin *et al.*, 1996; Kant *et al.*, 1997; Wehmann *et al.*, 1997). We describe here the differentiation of Indian pathotypes of NDV using RT-PCR and restriction enzyme digestion of the F gene sequence encoding the fusion protein cleavage site, with direct detection of virus in a clinical sample.

MATERIALS AND METHODS

Viruses

Four field isolates of NDV collected from various parts of India were used, including one isolate from a pigeon, one field isolate from Nepal and three vaccine strains. One clinical sample obtained as infected lung tissue was also included. Details of these viruses are given in Table I. All the viruses except TP1-98 from the clinical sample were propagated in 11-day-old embryonated chicken eggs (ECE).

Extraction of total RNA

The vaccine strain F, propagated in bulk in ECE, was purified by sucrose density gradient centrifugation, following the procedure described by Nagai and colleagues (1976) with some modifications. Briefly, the clarified virus was pelleted by centrifugation at 80 000*g* for 1 h through 30% sucrose. The reconstituted virus was further purified by ultracentrifugation in a 30% and 60% discontinuous sucrose gradient at 100 000*g* for 1 h. RNA extracted from the purified virus by guanidium thiocyanate was used to standardize the technique of RT-PCR.

Using the RNeasy kit (QIAGEN GmbH, Hilden, Germany), the total RNA from the infected allantoic fluid of all the isolates was extracted following the manufacturer's

TABLE I
Details of the Newcastle disease viruses used

No.	Isolates/ strains	MDT (h)	IVPI	ICPI	Pathotype	Origin
1	TN1-87 (chicken)	NT	NT	NT	Unknown	Tamil Nadu, India
2	UP2-92 (pigeon)	68	1.01	1.16	Mesogenic	Uttar Pradesh, India
3	NP1-93 (chicken)	48	2.21	1.65	Velogenic	Nepal
4	UP1-93 (chicken)	53	2.25	1.75	Velogenic	Uttar Pradesh, India
5	WB1-94 (chicken)	45	2.32	1.78	Velogenic	West Bengal, India
6	TP1-98 (chicken)	NT	NT	NT	Unknown	Clinical sample from Tripura, India
7	F	Standard lentogenic vaccine strain in use in India				
8	LaSota	Standard lentogenic vaccine strain in use in India				
9	R_2B	Standard mesogenic vaccine strain in use in India				

NT, not tested

protocol. For extraction of total RNA from a suspected clinical sample, TRIZOL reagent (Gibco-BRL, Gaithersburg, MD, USA) was used as described by the manufacturer. Total RNA was also extracted from uninfected allantoic fluid for use as a negative control.

Primers

Two pairs of primers were designed to amplify the F gene sequence encoding the fusion protein cleavage site. The primers were designed from the published sequence of the velogenic NDV strain Miyadera (Toyoda *et al.*, 1989), using the 'PrimerSelect' programme of 'Lasergene' software (DNASTAR Inc., Madison, WI, USA). Primer pair no. 1 was designed to amplify a 356 bp sequence (forward, 5′-GCAGCTGCAGG-GATTGTGGT-3′, nucleotide postion 158–177; reverse, 5′-TCTTTGAGCAGGAG-GATGTTG-3′, nucleotide position 513–493) and primer pair no. 2 to amplify a 216 bp nested sequence (forward, 5′-CCCCGTTGGAGGCATAC-3′, nucleotide position 282–298; reverse, 5′-TGTTGGCAGCATTTTGATTG-3′, nucleotide position 497–478).

Reverse transcription–polymerase chain reaction

RT-PCR was standardized using purified RNA of the F strain of NDV. For cDNA synthesis, 100 ng of purified viral RNA and 50 ng of random hexamer primers, in a 20 μl reaction mixture, were heated at 70°C for 15 min, followed by incubation at 20°C for

10 min. After adding 4 µl of 5× RT buffer (250 mmol/L Tris-HCl, pH 8.3, 375 mmol/L KCl, 15 mmol/L $MgCl_2$, 50 mmol/L DTT), 40 U RNasin (Promega, Madison, WI, USA), 500 µmol/L dNTPs (Boehringer Mannheim GmbH, Mannheim, Germany), 10 mmol/L DTT (Promega) and 200 U of MMLV-reverse transcriptase (Promega), the reaction was carried out at 37°C for 1 h, followed by inactivation of the enzyme at 95°C for 3 min.

PCR was carried out in a 50 µl reaction mixture using 15 pmol each of the forward and reverse primers of primer pair no. 1, 200 µmol/L dNTPs, 1× PCR buffer with 0.9 mmol/L $MgCl_2$, 6 µl cDNA and 3 U of *Taq* DNA polymerase (Promega). After denaturation of the template at 94°C for 2.5 min, amplification was carried out for 35 cycles of three steps, at 94°C, 58°C and 72°C, each for 45 s, with final extension at 72°C for 5 min. A nested PCR was performed on the PCR products, using primer pair no. 2 under the same reaction conditions, except that the Mg^{2+} concentration was 1.5 mmol/L and the annealing temperature was 56°C. This standardized technique of RT-PCR was applied to the total RNA extracted from the infected allantoic fluids and the clinical sample. The authenticity of the amplicons generated using primer no. 1 was confirmed by their size in 1.5% agarose gel and nested PCR.

Restriction enzyme analysis

Amplified products of 356 bp, generated by primer pair no. 1, were purified using the 'Wizard PCR Preps DNA Purification System' (Promega). Approximately 1 µg of the purified amplicons of all the isolates and vaccine strains were subjected to digestion with 10–15 U of the restriction enzymes *Alu*I, *Bgl*I, *Hae*III, *Hin*fI, *Hha*I, *Rsa*I and *Sty*I at 37°C overnight. Restriction digestion with *Taq*I restriction enzyme was carried out at 60°C overnight. The restriction enzymes were obtained from Promega.

RESULTS

The technique using RT-PCR to amplify the fusion protein cleavage site sequence was easily standardized on RNA from the purified F strain of NDV, as was evident from the generation of the specific amplicon of 356 bp size, which was also confirmed by the nested PCR, yielding product of 216 bp (Figure 1). The standardized RT-PCR was able to detect NDV in allantoic fluids infected with field isolates and vaccine strains using primer pair no. 1 (Figure 2) and by nested PCR with primer pair no. 2 (Figure 3).

Digestion with restriction enzyme *Bgl*I of the 356 bp PCR products of vaccine strains F, LaSota and two field isolates, UP2-92 of pigeon origin and TN1-87, yielded two fragments of approximately 240 bp and 116 bp. However, no restriction site was found in mesogenic strain R_2B or field isolates NP1-93, UP1-93 and WB1-94 (Figure 4). Another restriction enzyme, *Hha*I did not have any sites in field isolates NP1-93, UP1-93 and WB1-94, whereas there was a restriction site resulting in two visible bands with a similar pattern in field isolates TN1-87, UP2-92 and three vaccine strains (Figure 5).

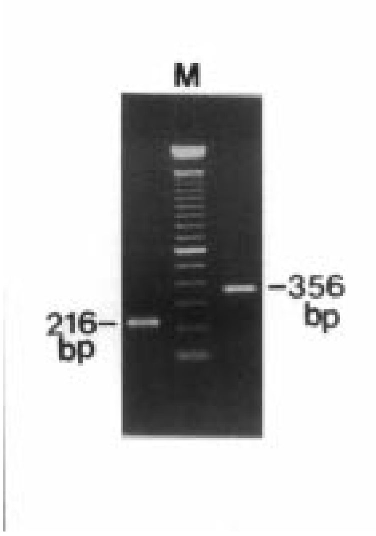

Figure 1. Agarose gel electrophoresis of 216 bp and 356 bp PCR products generated from RNA of purified NDV F strain along with 100 bp DNA ladder

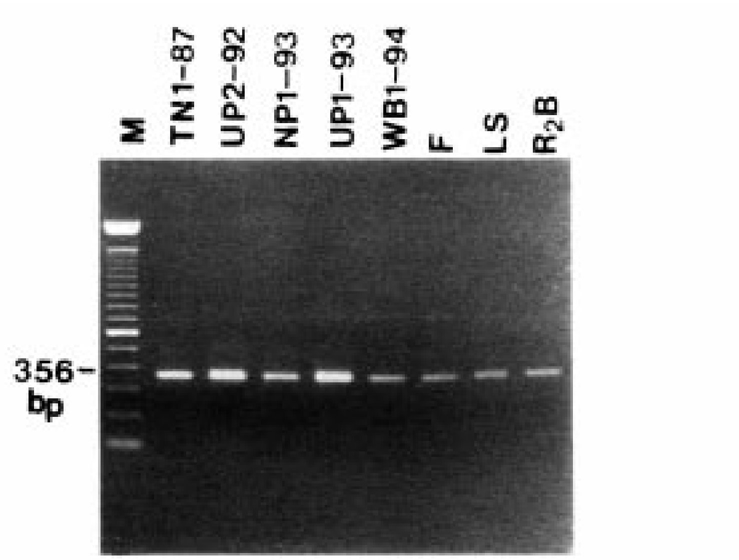

Figure 2. Agarose gel electrophoresis of 356 bp PCR products generated from field isolates and vaccine strains using primer pair no. 1. Lane M, ϕX174 DNA digested with *Hae*III as molecular size marker

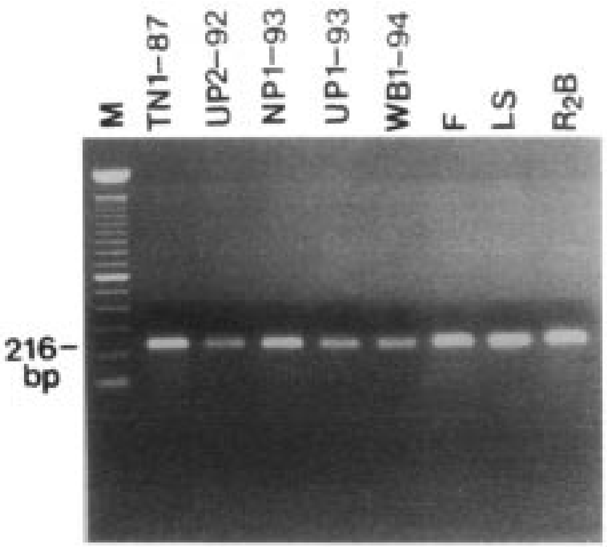

Figure 3. Agarose gel electrophoresis of 216 bp nested PCR products generated from field isolates and vaccine strains. Lane M, ϕX174 DNA digested with *Hae*III as molecular size marker

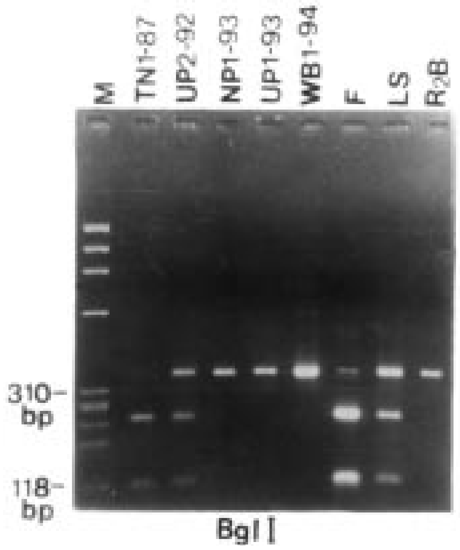

Figure 4. Electrophoresis of restriction enzyme *Bgl*I-digested 356 bp PCR product of field isolates and vaccine strains in 2% agarose gel. Lane M, ϕX174 DNA digested with *Hae*III as molecular size marker

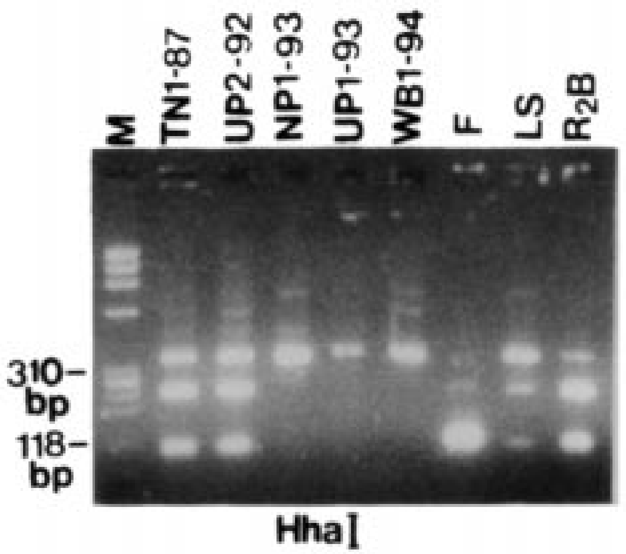

Figure 5. Restriction enzyme *Hha*I-digested 356 bp product of field isolates and vaccine strains in 2% agarose gel. Lane M, ϕX174 DNA digested with *Hae*III as molecular size marker

Three field isolates, NP1-93, UP1-93 and WB1-94, along with the mesogenic strain R_2B, gave a similar pattern with *Hin*fI restriction enzyme. Vaccine strain LaSota, pigeon isolate UP2-92 and field isolate TN1-87 had identical restriction profiles, which differed from those with the other three field isolates and the vaccine strain R_2B. However, the restriction pattern of the F strain with this enzyme was different from all other viruses (Figure 6). The restriction enzyme profile of *Alu*I for the mesogenic strain R_2B was similar to that of field isolates NP1-93 and UP1-93, whereas that of the vaccine strain LaSota was similar to that of field isolate TN1-87 and pigeon isolate UP2-92. However, the field isolate WB1-94 and vaccine strain F gave different restriction patterns (Figure 7).

Vaccine strain F and field isolate WB1-94 had no site for restriction enzyme *Hae*III, whereas there was partial cleavage with all other strains and isolates (Figure 8). Restriction enzyme *Sty*I had the same number of restriction sites in all the field isolates and vaccine strains (Figure 9), whereas *Taq*I and *Rsa*I enzymes had no sites in any of the field isolates or vaccine strains tested.

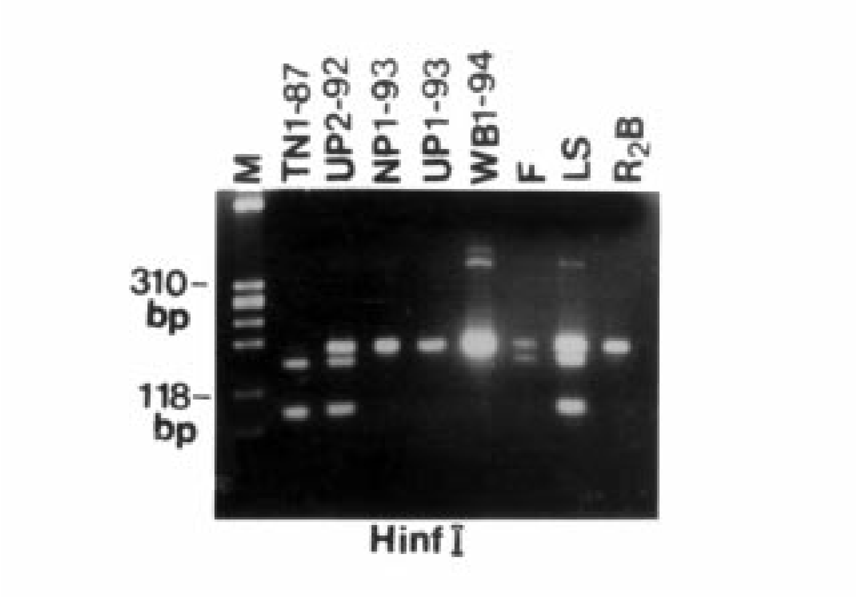

Figure 6. Electrophoresis of restriction enzyme *Hin*fI-digested 356 bp PCR product of field isolates and vaccine strains in 2% agarose gel. Lane M, ϕX174 DNA digested with *Hae*III as molecular size marker

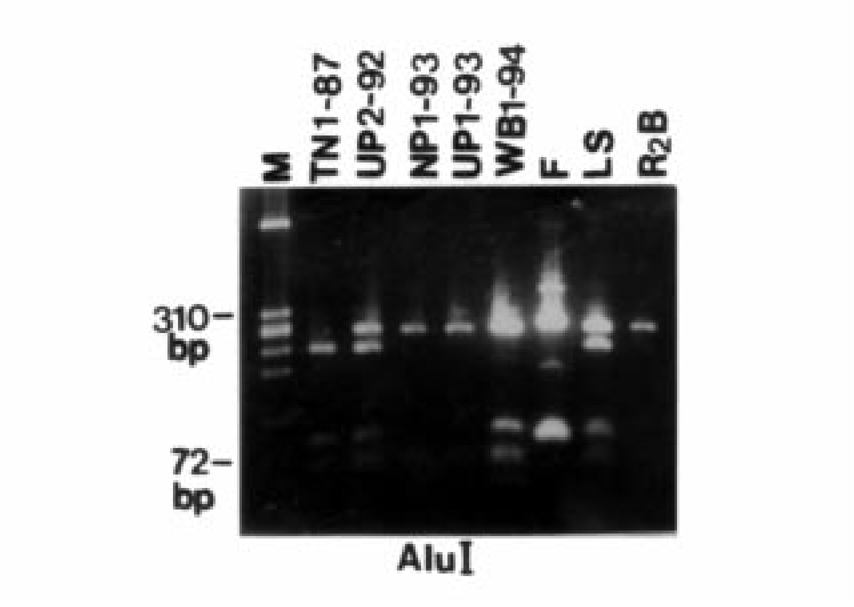

Figure 7. Restriction enzyme *Alu*I-digested 356 bp product of field isolates and vaccine strains in 2% agarose gel. Lane M, ϕX174 DNA digested with *Hae*III as molecular size marker

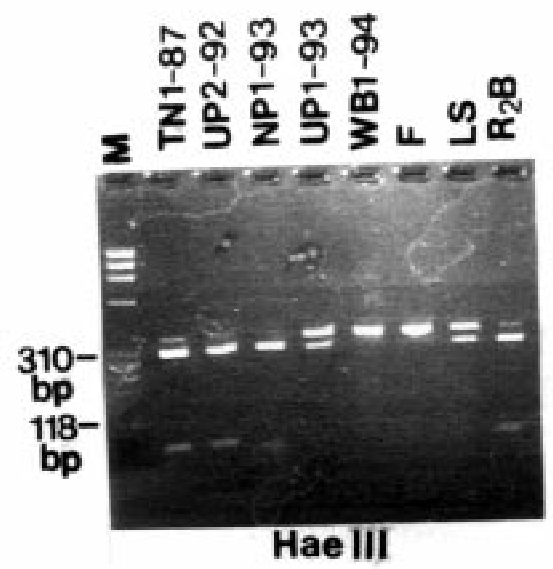

Figure 8. Restriction enzyme *Hae*III-digested 356 bp product of field isolates and vaccine strains in 2% agarose gel. Lane M, ϕX174 DNA digested with *Hae*III as molecular size marker

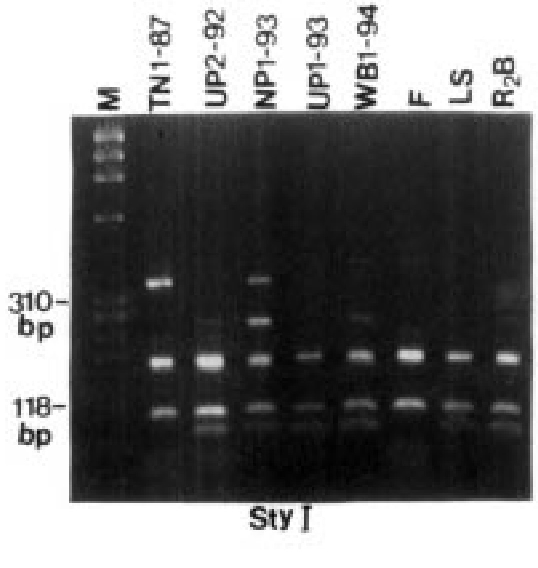

Figure 9. Electrophoresis of restriction enzyme *Sty*I-digested 356 bp PCR product of field isolates and vaccine strains in 2% agarose gel. Lane M, ϕX174 DNA digested with *Hae*III as molecular size marker

DISCUSSION

The technique of RT-PCR followed by restriction enzyme digestion has been exploited by many workers to differentiate the pathotypes of NDV (Ballagi-Pordany *et al.*, 1996; Kant *et al.*, 1997). In the present study, by changing the concentration of Mg^{2+} and the annealing temperature of the primers, a product of specific 356 bp size could be generated by RT-PCR from all the field isolates and vaccine strains. The authenticity of these primary amplicons was confirmed by agarose gel electrophoresis and nested PCR.

For restriction analysis, further purification of the 356 bp amplicons was found to be mandatory, as has also been reported by Jestin and Jestin (1991). In the present study, restriction enzyme digestion with *Bgl*I could differentiate the lentogenic strains (F and LaSota) from the mesogenic (R_2B) and velogenic strains (NP1-93, UP1-93 and WB1-94). The pigeon isolate, UP2-92, which had not been characterized as pigeon paramyxovirus-1, a variant of NDV, earlier reported to be mesogenic strain by IVPI, ICPI and MDT in chickens (Mishra, 1997) and a field isolate, TN1-87 (unknown pathotype), had the same restriction profile as the lentogenic strains. Collins and colleagues (1996) were unable to correlate pathotyping of pigeon paramyxovirus isolates by the conventional IVPI method and sequence analysis of the F gene at the cleavage site. Restriction enzyme *Hha*I could differentiate mesogenic and lentogenic strains from velogenic isolates. Therefore, based on restriction enzyme analysis with these two enzymes, it was possible to differentiate all three pathotypes of NDV, without any exceptions among viruses of the same pathotype.

Mesogenic and velogenic ND viruses have been reported to be similar at the nucleotide and amino acid levels in their fusion protein cleavage site (Collins *et al.*, 1993) and differentiation of these pathotypes by restriction enzyme analysis has not been reported earlier. The differences in the restriction enzyme profiles obtained in this study could be due to sequence variation in the F gene at other than the cleavage site. However, on the basis of the *Mbo*I and *Hin*fI restriction enzyme profiles of a RT-PCR amplified M gene sequence, Wehmann and colleagues (1997) were even able to differentiate two lentogenic strains, B_1 and LaSota. In this study, based on restriction profile of *Bgl*I and *Hha*I, the virus in a clinical sample was found to be lentogenic in type.

The restriction enzymes *Rsa*I, *Hin*fI, *Bst*OI and *Nar*I have been used by other workers to differentiate virulent and avirulent NDV isolates (Jestin and Jestin, 1991; Ballagi-Pordany *et al.*, 1996). We did not find any site for restriction enzyme *Rsa*I in any of the isolates or strains tested. This difference may have arisen because other workers analysed amplicons of 1349 bp rather than 356 bp, as used in this study. Field isolates NP1-93, UP1-93 and WB1-94 had a unique pattern for restriction enzyme *Hin*fI and so could be differentiated from the vaccine strains, field isolate TN1-87 and the pigeon isolate UP2-92. As the restriction enzyme profile with *Sty*I restriction enzyme was similar for viruses of all pathotypes, this enzyme could be used to characterize and confirm NDV isolates in general, without grouping them into different pathotypes.

The technique of RT-PCR, as standardized in this study, should be useful in directly

detecting NDV in clinical samples, without necessitating virus isolation in ECE, except where this is required by statutory bodies. It will also be useful for classifying NDV isolates of varying pathogenicity into velogenic, mesogenic and lentogenic strains, particularly by using restriction enzymes *Bgl*I and *Hha*I, so avoiding the use of more cumbersome and inhumane conventional methods. Sequence analysis of this portion of the F gene would further confirm these pathotypes and so facilitate the generation of molecular epidemiological data.

ACKNOWLEDGEMENTS

The authors thank the Director, Indian Veterinary Research Institute, Izatnagar, for providing the facilities, and Drs K.C. Verma and S.C. Mishra for providing virus isolates and strains.

REFERENCES

Alexander, D.J., 1990. Avian paramyxoviridae – recent developments. *Veterinary Microbiology*, **23**, 103–114

Alexander, D.J., 1991. Newcastle disease and other paramyxovirus infection. In: B.W. Calnek (ed.), *Diseases of Poultry*, (Iowa State University Press, Ames, IA), pp. 496–519

Alexander, D.J. and Allan, W.H., 1974. Newcastle disease virus pathotypes. *Avian Pathology*, **4**, 269–278

Ballagi-Pordany, A., Wehmann, E., Herczeg, J., Belak, S. and Lomniczi, B., 1996. Identification and grouping of Newcastle disease virus strains by restriction site analysis of a region from F gene. *Archives of Virology*, **141**, 243–261

Collins, M.S., Bashiruddin, J.B. and Alexander, D.J., 1993. Deduced amino acid sequences at the fusion protein cleavage site of Newcastle disease viruses showing variation in antigenicity and pathogenicity. *Archives of Virology*, **128**, 363–370

Collins, M.S., Strong, I. and Alexander, D.J., 1996. Pathogenicity and phylogenetic evolution of the variant Newcastle disease viruses termed "Pigeon PMV-1 virus" based on the nucleotide sequence of fusion protein gene. *Archives of Virology*, **141**, 635–647

Jestin, V. and Jestin, A., 1991. Detection of Newcastle disease virus RNA in infected allantoic fluids by *in-vitro* enzymatic amplification (PCR). *Archives of Virology*, **118**, 151–161

Kant, A., Koch, G., Roozelaar, D.J.V., Balk, F. and Huurne, A.T., 1997. Differentiation of virulent and non-virulent strains of Newcastle disease virus within 24 hours by polymerase chain reaction. *Avian Pathology*, **26**, 837–849

Marin, M.C., Villegas, P., Bennett, J.D. and Seal, B.S., 1996. Virus characterization and sequence of the fusion protein gene cleavage site of recent Newcastle disease virus field isolates from the South Eastern United States and Puerto Rico. *Avian Diseases*, **40**, 382–390

Mayo, M.A. and Pringle, C.R., 1998. Virus taxonomy – 1997. *Journal of General Virology*, **79**, 649–657

Mishra, S., 1997. *Studies on pathogenicity, strain differentiation and antigenic characterization of Newcastle disease virus isolated from different avian species*, (PhD thesis, Deemed University, Indian Veterinary Research Institute, Izatnagar, U.P., India)

Nagai, Y., Klenk, H.I. and Rott, R., 1976. Proteolytic cleavage of the viral glycoproteins and its significance for the virulence of Newcastle disease virus. *Virology*, **72**, 494–498

Seal, B.S., King, D.J. and Bennett, J.D., 1995. Characterization of Newcastle disease virus isolates by reverse transcription PCR coupled to direct nucleotide sequencing and development of sequence data base for pathotype prediction and molecular epidemiological analysis. *Journal of Clinical Microbiology*, **33**, 2624–2630

Toyoda, T., Sakaguchi, T., Imai, K., Inocencio, N.M., Gotoh, B., Hamaguchi, M. and Nagai, Y., 1987. Structural comparison of the cleavage-activation site of the fusion glycoprotein between virulent and avirulent strains of Newcastle disease virus. *Virology*, **158**, 242–247

Toyoda, T., Sakaguchi, T., Hirota, H., Gotoh, B., Kuma, K., Miyata, T. and Nagai, Y., 1989. Newcastle disease virus evolution II. Lack of gene recombination in generating virulent and avirulent strains. *Virology*, **169**, 273–282

Umino, Y., Kohma, T., Sato, T.A., Sugiura, A., Klenk, H.D. and Rott, R., 1990. Monoclonal antibodies to three structural proteins of Newcastle disease virus: biological characterization with particular reference to the conformational change of envelope glycoproteins associated with proteolytic cleavage. *Journal of General Virology*, **71**, 1189–1197

Wehmann, E., Herczeg, J., Ballagi-Pordany, A. and Lomniczi, B., 1997. Rapid identification of Newcastle disease virus vaccine strains LaSota and B-1 by restriction site analysis of their matrix gene. *Vaccine*, **15**, 1430–1433

(Accepted: 14 November 1999)
