# Supplementary material for: Newcastle disease burden in Nepal and efficacy of Tablet I2 vaccine in commercial and backyard poultry production
Source: PLoS One. 2023 Mar 10;18(3):e0280688. doi: 10.1371/journal.pone.0280688 (PMC10004539; doi:10.1371/journal.pone.0280688)
Supplement: Attachment — Submitted filename: Sequence_analysis_of_the_cleavage_site-encoding_re.pdf [file pone.0280688.s006.pdf]

ResearchGate

See discussions, stats, and author profiles for this publication at: https://www.researchgate.net/publication/23966617

Sequence analysis of the cleavage site-encoding region of the fusion protein gene of Newcastle disease viruses from India and Nepal

Article *in* Avian Pathology · January 2001

DOI: 10.1080/713651205 · Source: PubMed

CITATIONS
26

READS
63

6 authors, including:

Ashok K Tiwari
ICAR-Indian Veterinary Research Institute
265 PUBLICATIONS **2,666** CITATIONS
SEE PROFILE

Ranjit Singh Kataria
National Bureau of Animal Genetic Resources
251 PUBLICATIONS **1,971** CITATIONS
SEE PROFILE

Pankaj Bharti Goswami
Sardar Patel University
54 PUBLICATIONS **1,090** CITATIONS
SEE PROFILE

Some of the authors of this publication are also working on these related projects:

Project Viral Genes as Oncolytic Agents View project

Project Phenotypic and genetic characterization of buffalo populations of Odisha. View project

All content following this page was uploaded by Ashok K Tiwari on 27 April 2015.

The user has requested enhancement of the downloaded file.

Avian Pathology (2000) **29**, 603–607

Sequence analysis of the cleavage site-encoding region of the fusion protein gene of Newcastle disease viruses from India and Nepal

T. Nanthakumar[1], A. K. Tiwari[1]*, R. S. Kataria[1], G. Butchaiah[2], J. M. Kataria[3] & P. P. Goswami[1]

[1]*National Biotechnology Centre and* [3]*Division of Avian Diseases, Indian Veterinary Research Institute, Izatnagar–243 122, India, and* [2]*Dean's Office, Rajiv Gandhi College of Veterinary and Animal Sciences, Pondicherry 605 009, India*

Five field isolates of Newcastle disease virus, including one from a pigeon from the Indian subcontinent, along with three vaccine strains have been characterized by sequence analysis of the fusion protein (F) gene in the region encoding the F_2–F_1 cleavage site. Based on the amino acid sequence present at the cleavage site and on the percent divergence at nucleotide and amino acid levels, three field isolates could be classified as velogenic and two were of lentogenic pathotypes. The velogenic pathotypes had the sequence RRQK/RRF at the cleavage site, while the lentogenic strains had GRQA/GRL at the corresponding position.

Introduction

Newcastle disease is a highly contagious viral disease of poultry, affecting almost all species of birds of different age groups worldwide. Depending upon the pathotype involved and susceptibility of the flock, the virus causes mortality ranging from 0 to 100%. The causative agent of the disease, Newcastle disease virus (NDV), has been classified as avian paramyxovirus–1 in the genus *Rubulavirus* of the family *Paramyxoviridae*. NDV has a negative-sense RNA genome of approximately 15 kb encoding six major proteins, fusion (F), haemagglutinin-neuraminidase (HN), matrix (M), phosphoprotein (P), RNA polymerase (L) and nucleoprotein (NP) (Mayo & Pringle, 1998). The fusion protein encoded by the F gene is synthesized as a F_0 precursor, which is activated only after cleavage into F_1 and F_2 subunits by host cell proteases (Nagai *et al.*, 1976; Umino *et al.*, 1990). The F protein is responsible for initiation of infection by fusion of the virion membrane with the host cell membrane and penetration of the virus into the cell (Marin *et al.*, 1996). Depending upon the virulence, ND viruses have been classified into less virulent lentogenic, moderately virulent mesogenic and highly virulent velogenic strains, by conventional methods of mean death time (MDT), intracerebral pathogenicity index (ICPI) and intravenous pathogenicity index (IVPI) (Alexander & Allan, 1974). More reliable, molecular techniques like reverse transcriptase-polymerase chain reaction (RT-PCR), restriction enzyme analysis and sequencing of different genes have been employed in the recent past to differentiate viruses of varying virulence (Ballagi-Pordany *et al.*, 1996; Collins *et al.*, 1996, 1998; Kant *et al.*, 1997; Werner *et al.*, 1999; Nanthakumar *et al.*, 2000). At the fusion protein cleavage site, the presence of a number of basic amino acids determines the pathogenicity of ND viruses. The presence of two pairs of basic amino acids at 112, 113 and 115, 116, along with phenylalanine at 117, makes the fusion protein of velogenic viruses more susceptible to cleavage by ubiquitous proteases, helping them to spread throughout the host. In contrast, the presence of two single basic amino acids at 113 and 116, along with leucine at 117, allows the fusion protein of avirulent

* To whom correspondence should be addressed. E-mail: aktiwari@ivri.up.nic.in
Received 14 April 2000. Accepted 17 July 2000.

ISSN 0307-9457 (print)/ISSN 1465-3338 (online)/00/060603-05 © 2000 Houghton Trust Ltd
DOI: 10.1080/03079450020016000

Table 1. *Details of the Newcastle disease viruses from India and Nepal*

Isolates/strains	Pathotype[a]	Origin	EMBL accession number/reference
TN 1–87	Unkown	Tamil Nadu, India	AJ249528
UP 2–92 (pigeon)	Mesogenic	Uttar Pradesh, India	AJ245812
NP 1–93	Velogenic	Nepal	AJ249526
UP 1–93	Velogenic	Uttar Pradesh, India	AJ249529
WB 1–94	Velogenic	West Bengal, India	AJ249530
F	Lentogenic	Vaccine strain in use in India	AJ249516
LaSota	Lentogenic	Vaccine strain in use in India	AJ249525
R_2B	Mesogenic	Vaccine strain in use in India	AJ249527
Miyadera	Velogenic	Japan	M18456
Australia-Victoria	Velogenic	Australia	M21881
Texas-US	Velogenic	USA	M33855
1168/84 (Pigeon)	Velogenic	UK	Collins *et al.* (1996)
Beaudette C	Mesogenic	USA	X04719
ULS–67	Lentogenic	Northern Ireland	M24694

[a] Determined by IVPI, ICPI and MDT.

NDV isolates to be cleaved only by trypsin-like proteases present mainly in the respiratory and digestive tracts (Collins *et al.*, 1996).

In this paper, we describe the characterization of ND viruses from India and Nepal by sequence analysis of part of the fusion protein gene encoding the cleavage site for pathotype prediction and molecular epidemiology.

Materials and Methods

The viruses (details presented in Table 1) used in this study were propagated in 11-day-old embryonated chicken eggs.

Total RNA extracted from 200 μl infected allantoic fluid using the RNeasy kit (QIAGEN) as described by the manufacturer was eluted with 30 μl nuclease free water. For cDNA synthesis, the protocol described earlier (Nanthakumar *et al.*, 2000) was followed. Briefly, approximately 4 μg heat-denatured total RNA and 50 ng random hexamer primers in a 20 μl reaction mixture were incubated at 37°C for 1 h, after adding 4 μl of 5 × RT buffer, 40 U RNasin (Promega), 500 μM dNTPs (Boehringer Mannheim), 10 mM di-thiothreitol (Promega) and 200 U MMLV-reverse transcriptase (Promega).

For PCR, 6 μl cDNA was amplified using 15 pmol each primer (forward, 5′-GCAGCT GCAGGGATTGTGGTG-3′, nucleotide positions 158–177; reverse, 5′-TCTTTGAGCAGG AGGATGTTG-3′, nucleotide positions 513–493) designed from published sequence of Japanese velogenic strain, Miyadera (Toyoda *et al.*, 1987). The amplification was carried out in a thermal cycler (PTC 200; MJ Research, USA), by initial denaturation at 94°C for 2.5 min followed by 35 cycles of 94, 58 and 72°C each for 45 s. The amplified products were analyzed on 1.5% agarose gel and purified through QIAquick PCR purification columns (QIAGEN).

Purified amplicons of the field isolates and vaccine strains were subjected to cycle sequencing using the ‘fmol’ DNA Sequencing System (Promega) and ^{35}S dATP following het direct incorporation method as described by the manufacturer. Nested primers (forward, 5′-CCCCGTTG GAGGCA TAC-3′, nucleotide positions 282–298; reverse, 5′-TGTTGGCAGCATTTTGATTG-3′, nucleotide positions 497–478) were used to sequence amplicons from both ends with the cycling conditions of 94°C for 2 min followed by 30 cycles of 94°C for 30 s, 42°C for 30 s and 70°C for 1 min. After running the samples on a polyacrylamide sequencing gel (Model S2; Life Technologies, Inc, USA), nucleotide sequences were read manually. These sequences are available in the EMBL database under the accession numbers presented in Table 1. For comparison, nucleotide and amino acid sequences of known reference strains, retrieved from the EMBL database, are also included (Table 1).

All the nucleotide and deduced amino acid sequences were aligned separately using the Clustal method in the MegAlign programme of the Lasergene software (DNASTAR Inc., USA).

Results

Nucleotide sequences of all the viruses from positions 197 to 507 (Toyoda *et al.*, 1987) and deduced amino acid sequences (position 51 to 153) were analyzed. The aligned, deduced amino acid sequences are shown in Figure 1. The total number of nucleotide differences among all the field isolates and vaccine strains ranged from 19 (UP2–92) to 25 (LaSota), whereas differences in amino acid were between 2 (R_2B) and 7 (LaSota) from the consensus. The nucleotide changes at position 287, in the field isolate UP1–93, and at positions 205, 226 and 274, in vaccine strain F, were specific to these viruses. All the three vaccine strains and two isolates, TN1–87 and UP2–92, had nine nucleotide differences in common but only two resulted in change of amino acids: 69M to L and 82E to D. Although there were a total of 17 changes at the nucleotide level in three velogenic field isolates (NP1–93, UP1–93 and WB1–94), only four at positions 357, 365, 407 and 416 resulted in changes of amino acids 104E to G, 107T to S, 121I to V and 124G to S, respectively.

The vaccine strains F, LaSota and the Indian Pigeon isolate UP2–92 had the amino acid sequence GRQGRL but field isolate TN1–87 had GRQARL at the cleavage site (112 to 117). Mesogenic strain R_2B and field isolates NP1–93

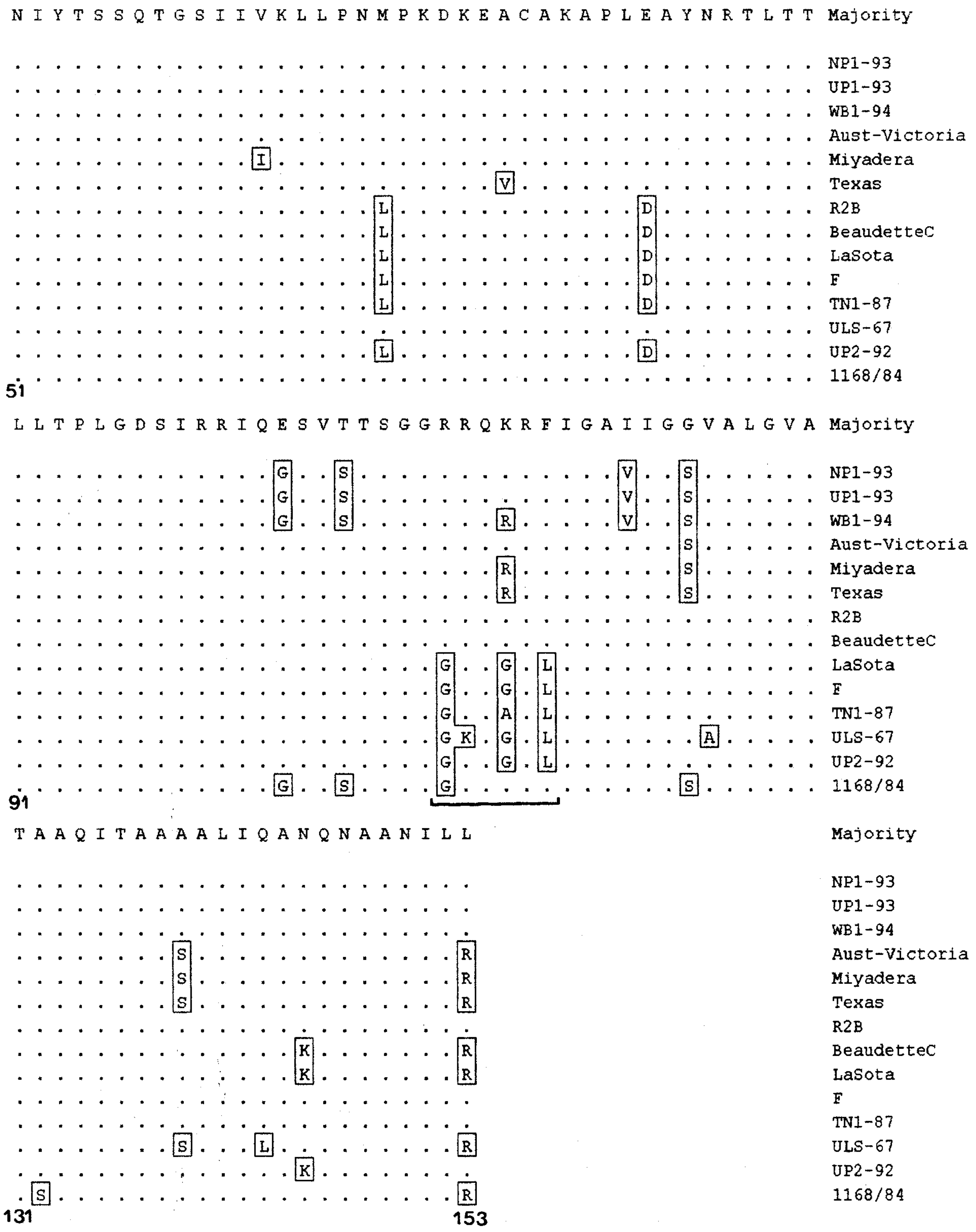

Figure 1. *Deduced amino acid sequence of NDV isolates from India (TN1–87, UP2–92, UP1–93 and WB1–94,) and Nepal (NP1–93), along with vaccine strains (R_2B, LaSota and F) and reported viruses of different pathotypes aligned by the Clustal method using the MegAlign program of Lasergene software (Amino acid positions as per Toyoda* et al. *(1987). Amino acid residues different from the consensus are boxed. The fusion protein cleavage site (112 to 117) is underlined.*

and UP1–93 had the sequence RRQKRF, whereas field isolate WB1–94 had RRQRRF at corresponding positions.

The divergence among three field isolates, UP1–93, NP1–93 and WB1–94, ranged from 1.3 to 2.6% at nucleotide level, and 0 to 1% at amino acid level. Among all other viruses, there was 1 to 6.1% divergence at nucleotide level and 1 to 5% at amino acid levels from the consensus. Mesogenic strain R_2B had 1.6% nucleotide and 2% amino acid

divergence from another reference mesogenic strain, Beaudette C, whereas its divergence was 14.9 to 15.7% (nucleotide) and 6.1 to 7.1% (amino acid) from Indian field isolates UP1–93, NP1–93 and WB1–94.

Discussion

Sequence analysis of the F gene encoding the fusion protein cleavage site has been exploited by many workers for the pathotype characterization of ND viruses (McGinnes & Morrison, 1986; Sato *et al.*, 1987; Toyoda *et al.*, 1987). In the present study, none of the field isolates and vaccine strains had 100% nucleotide homology with other viruses of the same pathotype. The Indian NDV field isolate UP1–93 and Nepalese isolate NP1–93 were 100% similar at amino acid level, which could be due to their close geographical origin. Stram *et al.* (1998) used percent divergence/similarity of F gene sequence for classifying ND viruses of different pathotypes. Similarly, in this study, based on percent divergence, pigeon isolate UP2–92 and field isolate TN1–87, along with vaccine strains F and LaSota, could be grouped as lentogenic pathotypes, R_2B as mesogenic and other three field isolates as a velogenic pathotype. Based on sequence of fusion protein, none of the field isolates tested was found to be of mesogenic pathotype.

The amino acid change 124G to S in the N terminal region of the F_1 protein and two pairs of basic amino acids at the cleavage site of the fusion protein, reported in reference velogenic strains (McGinnes & Morrison, 1986; Toyoda *et al.*, 1987; Seal *et al.*, 1995; Collins *et al.*, 1996), were also present in the field isolates NP1–93, UP1–93 and WB1–94 characterized, confirming them to be of velogenic type. By pathogenicity studies using MDT, ICPI and IVPI, and also by RT-PCR/ restriction enzyme analysis of same region of F gene, these Indian and Nepalese NDV field isolates have already been reported to be of velogenic pathotypes (Nanthakumar *et al.*, 2000). The presence of single basic amino acid at cleavage site was characteristic of lentogenic viruses and RRQKRF along with amino acid S at position 124, similar to reported mesogenic strain Beaudette C, reconfirmed R_2B as mesogenic pathotype. The Indian pigeon isolate UP2–92, earlier reported to be mesogenic type by MDT, ICPI and IVPI (Mishra, 1997), had amino acid changes at cleavage site characteristic of lentogenic ND viruses. Earlier, Stram *et al.* (1998), on the basis of amino acid changes, grouped two Israeli isolates as velogenic pathotypes that they could not classify by MDT and ICPI due to inconsistent results.

The fusion-inducing hydrophobic stretch at the N terminus of the F_1 protein (117 to 142 amino acid residues), cysteine at position 76, important in disulphide bond formation between F_1 and F_2, and amino acids at potential glycosylation site (85-NRT–87) were found to be conserved in all the ND viruses irrespective of pathotype, as reported earlier by other workers (Toyoda *et al.*, 1987; Seal *et al.*, 1995). All these regions could be important for exact conformation of fusion protein vis-à-vis survival of the virus in nature.

Sequence analysis of larger gene fragments of a greater number of field isolates from this part of the world would provide further information on pathotypic characteristics of prevalent ND viruses, which could be useful in evolving better prophylactic measures.

Acknowledgements

The authors wish to thank the Director IVRI, Izatnagar, for providing necessary facilities to carry out the work, Mr. N. Prasad for technical assistance and Dr M. A. Skinner of IAH, Compton, UK, for assistance with the manuscript.

References

Alexander, D.J. & Allan, W.H. (1974). Newcastle disease virus pathotypes. *Avian Pathology, 4*, 269–278.

Ballagi-Pordany, A., Wehman, E., Herczeg, J., Belak, S. & Lomniczi, B. (1996). Identification and grouping of Newcastle disease virus strains by restriction enzyme site analysis of a region from the F gene. *Archives of Virology, 141*, 243–261.

Collins, M.S., Strong, I. & Alexander, D.J. (1996). Pathogenicity and phylogenetic evaluation of Newcastle disease viruses termed 'pigeon PMV–1viruses' based on the nucleotide sequence of fusion protein gene. *Archives of Virology, 141*, 635–647.

Collins, M.S., Franklin, S., Strong, I, Meulemans, G. & Alexander, D.J. (1998). Antigenic and phylogenetic studies on a variant Newcastle disease virus using anti-fusion protein monoclonal anitbodies and partial sequencing of the fusion protein genes. *Avian Pathology, 27*, 90–96.

Kant, A., Koch, G., van Roozelaar, D.J., Balk, F. & Huurne, A.T. (1997). Differentiation of virulent and non virulent strains of NDV with in 24 hours by polymerase chain reaction. *Avian Pathology, 26*, 837–849.

Marin, M.C., Villegas, P., Bennett, J.D. & Seal B.S. (1996). Virus characterisation and sequence of the fusion protein gene cleavage site of recent Newcastle disease virus field isolates from the South Eastern United States and Puerto Rico. *Avian Diseases, 40*, 382–390.

Mayo, M.A. & Pringle, C.R. (1998). Virus Taxonomy – 1997. *Journal of General virology, 79*, 649–657.

McGinnes, L.W. & Morrison, T.G. (1986). Nucleotide sequence of the gene encoding the Newcastle disease virus fusion protein and compasions of paramyxovirus fusion protein sequences. *Virus Research, 5*, 343–356.

Mishra, S. (1997). Studies on pathogenicity, strain differentiation and antigenic characterization of Newcastle disease viruses isolated from different avian species. Ph.D. thesis. Izatnagar, UP, India: Indian Veterinary Research Institute.

Nagai, Y., Klenk, H.-D. & Rott, R., (1976). Proteolytic cleavage of the viral glycoproteins and its significance for their virulence of Newcastle disease virus. *Virology, 72*, 494–508.

Nanthakumar, T., Kataria, R.S., Tiwari, A.K., Butchaiah, G. & Kataria, J.M. (2000). Pathotyping of Newcastle disease viruses by RT-PCR and restriction enzyme analysis. *Veterinary Research Communication, 24*, 275–286.

Sato, H., Oh-hira, M., Ishida, N., Imamura, Y., Hattori, S. & Kawakita, M. (1987). Molecular cloning and nucleotide sequence of P, M and F genes of Newcastle disease virus avirulent strain D26. *Virus Research, 7*, 241–255.

Seal, B.S., King, D.J. & Bennett, J.D. (1995). Characterization of Newcastle disease virus isolates by reverse transcription PCR coupled to direct nucleotide sequencing and development of sequence data base for pathotype prediction and molecular epidemiological analysis. *Journal of Clinical Microbiology, 33*, 2624–2630.

Stram, Y., Schlri, D., Chinitch, Y., David, D., Molad, T. & Samina, T. (1998). Molecular characterization of an unassigned Israeli Newcastle disease virus isolate. *Avian Diseases, 42*, 746–751.

Toyoda, T., Sakaguchi, T., Imai, K., Inocencio, N.M., Gotoh, B., Hamaguchi, M. & Nagai, Y. (1987). Structural comparison of the cleavage-activation site of the fusion glycoprotein between virulent and avirulent strains of Newcastle disease virus. *Virology, 158*, 242–247.

Umino, Y., Kohama, T., Sato, T.A., Sugiura, A., Klenk, H.-D. & Rott, R. (1990). Monoclonal antibodies to three structural proteins of Newcastle disease virus: biological characterization with particular reference to the conformational change of envelope glycoproteins associated with proteolytic cleavage. *Journal of General Virology, 71*, 1189–1197.

Werner, O., Römer-Oberdörfer, A., Köllner, B., Manvell, R.J. & Alexander, D.J. (1999). Characterization of avian paramyxovirus type 1 strains isolated in Germany during 1992 to 1996. *Avian Pathology, 28*, 79–88.

RÉSUMÉ

Analyse de la séquence du gène codant pour la protéine de fusion, au niveau du site de clivage, de virus de la maladie de Newcastle isolés en Inde et au Népal

Cinq isolats du virus de la maladie de Newcastle incluant une souche de pigeon du sous continent indien et trois souches vaccinales ont été caracterisés par l'analyse de la séquence du gène de la protéine F de fusion, au niveau de la région codant pour le site de clivage F_2–F_1. Basé sur la séquence en acides aminés présents au site de clivage et sur le pourcentage de divergence au niveau des nucléotides et des acides aminés, trois des isolats ont pu être classés comme étant des pathotypes vélogènes et deux des lentogènes.

Les pathotypes vélogènes présentaient la séquence RRQK/RRF au niveau du site de clivage alors que les souches lentogènes présentaient la séquence GRQA/GRL.

ZUSAMMENFASSUNG

Sequenzanalyse der die Spaltungsstelle kodierenden Region des Fusionsprotein-Gens von Newcastle-Viren aus Indien und Nepal

Fünf Feldisolate des Virus der Newcastle-Krankheit, darunter eins von einer Taube vom indischen Subkontinent, wurden zusammen mit drei Vakzinestämmen durch die Sequenzanalyse des Fusionsprotein (F)-Gens in der Region charakterisiert, die die F_2–F_1-Spaltungsstelle kodiert. Auf der Grundlage der Aminosäuresequenz an der Spaltungsstelle und der prozentualen Abweichung bei Nukleotiden und Aminosäuren konnten drei der Feldisolate als velogen charakterisiert werden und zwei waren lentogene Pathotypen. Die velogenen Pathotypen hatten an der Spaltungsstelle die Sequenz RRQK/RRF, während die lentogenen Stämme an der entsprechenden Stelle GRQA/GRL hatten.

RESUMEN

Análisis de la secuencia de la región del gen que codifica para el punto de escisión de la proteína de fusión de los virus de la enfermedad de Newcastle de la India y el Nepal

Se analizaron las secuencias de la región del gen de la proteína de fusión (F) que codifica para el punto de escisión de F_2–F_1 de cinco cepas de virus de la enfermedad de Newcastle, incluída una cepa de paloma que provenía del subcontinente indio, junto con tres cepas vacunales. En base a la secuencia de aminoácidos del punto de escisión y al porcentaje de divergencia a nivel de nucleótidos y aminoácidos, se clasificaron las tres cepas de campo como velogénicas y las otras dos resultaron de patotipo lentogénico. Los patotipos velogénicos presentaron la secuencia RRQK/RRF en el punto de escisión mientras que las cepas lentogénicas presentaron la secuencia GRQA/GRL en la posición correspondiente.
