# Supplementary material for: Newcastle disease burden in Nepal and efficacy of Tablet I2 vaccine in commercial and backyard poultry production
Source: PLoS One. 2023 Mar 10;18(3):e0280688. doi: 10.1371/journal.pone.0280688 (PMC10004539; doi:10.1371/journal.pone.0280688)
Supplement: Attachment — Submitted filename: VaccinationAkeywaytopreventNewcastleDiseaseInPoultryofNepal.pdf [file pone.0280688.s007.pdf]

ResearchGate

See discussions, stats, and author profiles for this publication at: https://www.researchgate.net/publication/339905748

Vaccination: A key way to prevent Newcastle disease in poultry of Nepal

Article · January 2020

DOI: 10.22271/veterinary.2020.v5.i1b.237

CITATIONS
0

READS
1,190

2 authors, including:

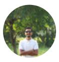

Uddab Poudel
Institute of Agriculture and Animal Science

9 PUBLICATIONS **44** CITATIONS

SEE PROFILE

All content following this page was uploaded by Uddab Poudel on 13 March 2020.

The user has requested enhancement of the downloaded file.

International Journal of Veterinary Sciences and Animal Husbandry

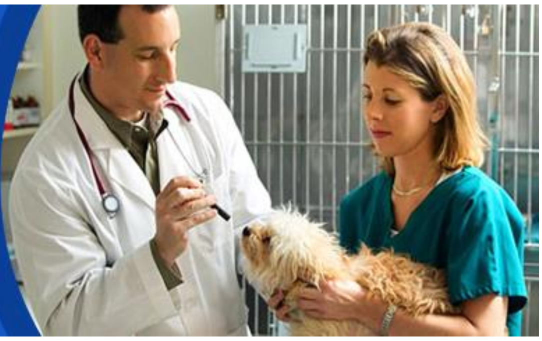

ISSN: 2456-2912
VET 2020; 5(1): 104-107
© 2020 VET
www.veterinarypaper.com
Received: 16-11-2019
Accepted: 20-12-2019

U Poudel
Internee, B.V.Sc. & A.H., Paklihawa Campus, Institute of Agriculture and Animal Science, Tribhuvan University, Siddharthanagar-1, Rupandehi, Nepal

U Dahal
Chief veterinary officer of national vaccine production laboratory, Tripureshwor, Kathmandu, Nepal

Corresponding Author:
U Poudel
Internee, B.V.Sc. & A.H., Paklihawa Campus, Institute of Agriculture and Animal Science, Tribhuvan University, Siddharthanagar-1, Rupandehi, Nepal

Vaccination: A key way to prevent Newcastle disease in poultry of Nepal

U Poudel and U Dahal

Abstract
Nepal is a country with variation in topography and diverse climatic zones. Agriculture is a major occupation for majority of Nepalese engaging 66% of total population in farming and Poultry farming is integral part of the agriculture in Nepal. Nepal has long history of traditional poultry keeping. Poultry are the important source of valuable dietary proteins and rural cash income. In recent data, about 72 million chickens and 400 thousand ducks are present which include commercial, small holder and backyard type of farm. Poultry sector is an emerging industry engaging and employing 70,000 people directly contributing about 4% in National GDP. In present days, the traditional poultry farming is replaced by commercial farming. But the challenge and constraints on poultry sector are increasing day by day. Outbreak and spread of emerging infectious disease is headache in poultry farming. Various disease like Avian influenza, Newcastle disease, IBD, IB, ILT, coccidiosis, Avian Mycoplasmosis, Fowl typhoid, Fowl pox etc. cause serious economic loss in poultry industry. Newcastle disease is common and spread throughout the country having higher incidence in the summer which is caused by avian paramyxovirus type-1 of Paramyxoviridae. Vaccines are important tool for prophylactic measures. In many countries, vaccination against ND is routinely practiced for prevention and control of disease and types of vaccines and vaccination schedule vary according to potential threat and virulence property of the field challenge virus. There is big challenge for vaccine researchers, manufacturers and scientists for development of effective vaccine against Newcastle disease. In this paper, we will discuss about present status of Newcastle disease and Vaccine produced in Nepal and Vaccination protocol to prevent spread of Newcastle Disease in Nepal.

Keywords: Commercial, vaccination, poultry, newcastle disease

Introduction
Nepal is an agriculture country with full of natural beauty with traces of artifacts lies between two giant country of the world, India in the East, South and West and China in the North where more than 66% population are directly engaged in farming [1]. Poultry farming is important sector contributing about 4% of National GDP in Nepal. Because of easy rearing, low investment cost and less manpower requirement, majority of population are attracted towards poultry industry. In past Bhramin society did not accept rearing of poultry, consumption of poultry meat and eggs were restricted and considered as taboo in Bhramin society of Nepal. In present days Poultry is widely accepted and useful addition to food menu. According to Poultry statistics 2071/2072, Commercial poultry farming was practiced in 64 districts out of 75 total districts and there were 21,956 farms present in these 64 districts of which 20,483 (93.29%) was broiler farm which has highest number, layers farm comes in second which was 1337 about 6.09%, hatchery was 128 which was about 0.58% and 8 (0.04%) Giriraj/ Koiler farm. The total bird population was 6,08,26,880 in which 5,26,66,029 were broilers, 69,08,595 layers and 12,52,255 other breed [2]. The number of chicken in 2072/2073 was increased by 12.8% with number of 6,86,30,638, which was further increased by 2% in 2073/74 and further increased by 3.1% in 2074/2075 with total of 7,22,45,732. The total duck population in 2072/2073 was 3,72,255 which was increased by 0.6% in 2073/2074 and further increased by 2.5% with total number of 4,04,670 duck population in 2074/2075 .The total meat production was 3,46,179 metric ton in which 18% of total consumed meat was contributed by poultry sector where chicken contribute 17.36% and Duck contribute 0.08%. The total egg production in 2074/2075 was 1,51,22,65,000 in which 99.05% was contributed by chicken eggs and 0.941% was contributed by Duck eggs [3]. In an average a Nepali used to eat 4.1kg per chicken and 44 eggs annually which is very below the global average,

showing greater scope of poultry farming in Nepal [4]. Poultry sector is emerging as a strong industry in Nepal which directly employing and engaging about 70,000 people. But, the poultry sector is challenged by many constraints in which spread and outbreak of disease in periodic interval is major one. There are various economic disease that are spread in the poultry of Nepal. *Viz*: Avian Influenza, Infectious Bursal Disease, Newcastle Disease, Infectious Bronchitis, Fowl pox, Avian Colibacillosis, Egg Drop Syndrome, Coccidiosis. Newcastle Disease is one of the important poultry disease in Nepal and is a worldwide problem which presents primarily as an acute respiratory disease, but depression, nervous manifestations, or diarrhea may be the predominant clinical form [5].Vaccination is best way to control and prevent Newcastle disease in Poultry [6]. Vaccination is excellent way to lessen clinical signs of infection caused by virulent ND virus in poultry [13].

Newcastle disease and ND virus

Newcastle Disease is a highly contagious, rapidly spreading viral infection that directly affects various species of both domesticated and wild birds to varying degrees. The most susceptible species are domestic fowl, turkeys, pigeons and parrots while the mild form of the disease affects various birds like ducks, geese, pheasants, quail and guinea fowl. The clinical sign of the disease result in various system including digestive, respiratory and nervous system, which range from a mild, almost inapparent respiratory disease to very severe depression, drop in egg production, increased respiration, profuse diarrhoea finally collapse, or long-term nervous signs will be seen in those birds which are survived. The disease is highly fatal in the severe form [7].

The etiology of Newcastle disease is Virulent strain of avian paramyxo virus type1 (APMV-1) of genus Avulavirus belonging to subfamily Paramyxovirinae and family Paramyxoviridae [8]. By serological testing and phylogenetic analysis, there are 10 serotypes of Avian paramyxovirus APMV-1 to APMV- 10 [9]. ND virus designated to APMV-1. ND virus is able to infect more than 200 species of birds but its severity vary with host and strain of the virus. On the basis of clinical signs the strain of ND virus is classified into five pathotype [10].

Table 1: Strain of ND virus with clinical signs

Strain of virus	Clinical signs
Viscerotropic Velogenic Strain	Causes high mortality, highly pathogenic form causing hemorrhagic intestinal lesion
Neurotropic Velogenic Strain	Causes high mortality, high virulence, following respiratory and nervous sign
Mesogenic Strain	Moderate virulence, Mortality up to 50%, usually respiratory sign, occasionally nervous signs
Lentogenic Strain	Cause low mortality, low virulence, Mild or subclinical respiratory infection seen

Status of Newcastle disease in Nepal

Various serological, Clinical and Pathological studies proved that Newcastle disease is prevalent in Nepal from many years ago. Prevalence of Newcastle disease is high in rural area due to poor supply of vaccines, not proper biosecurity in farm and reoccurrence of disease is frequently encountered. Recent data of 2018 revels 90 outbreak of Newcastle Disease in which Province number 1,2 and 5 have maximum outbreak of 27,15 and 25 respectively. Around nine hundred thousand birds were susceptible in which 74,986 birds were found affected and 7,363 were died from Newcastle disease same year and around 4,19,052 birds were vaccinated and 67,623 birds were treated [11].

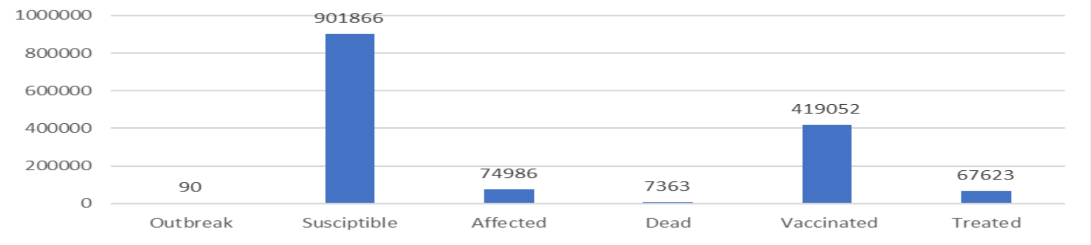

Figure 1: Overall situation of Newcastle Disease in 2018

Source: Annual Epidemiological Bulletin January- December 2018 [11

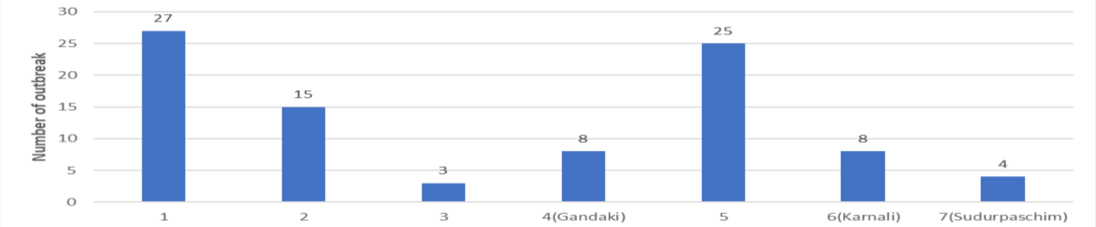

Figure 2: Province wise distribution of outbreaks of ND during Jan- December 2018

Source: Annual Epidemiological Bulletin January- December 2018 [11]

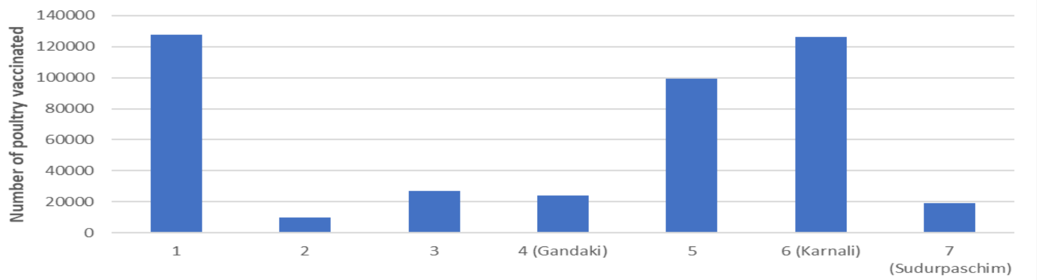

Figure 3: Province wise vaccination against ND For disease Control

Source: Annual Epidemiological Bulletin January- December 2018 [11]

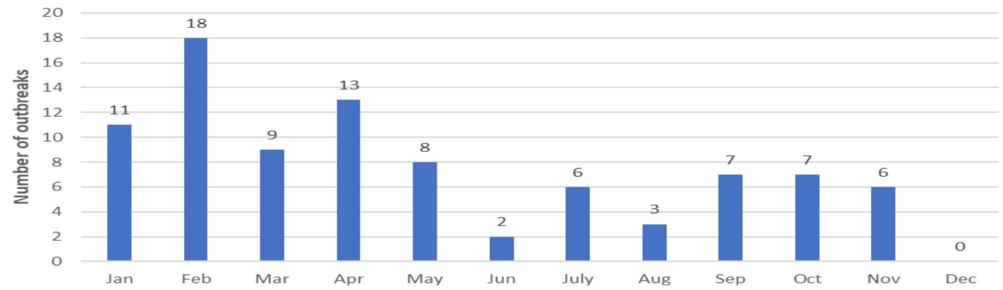

Figure 4 : Temporal distribution of outbreak of ND during Jan- december,2018

Source: Annual Epidemiological Bulletin January- December 2018 [11]

History of newcastle disease vaccine production in Nepal

In 1968, Poultry vaccine against Newcastle disease were produced by Central Biological Production Laboratory to protect poultry population. That time ND F- strain and ND R2B strain vaccine were introduced. The another strain of Newcastle disease La Sota strain vaccine was produced in 1998 to protect from Velogenic strain of ND virus, Vaccine was popular for its easy administration as it does not require vaccinator and can be given in drinking water. Earlier poultry vaccines were produced from fertile eggs available in the local hatchery but from 1997 the production of poultry vaccines were started by using Specific pathogen free eggs. Focusing for backyard poultry and village chickens, the production of heat stable vaccine against ND i.e. ND I2 was started from 2008 [12].

Prophylactic measure

Newcastle disease is major worldwide problem in the poultry industry. Many country routinely practiced ND vaccination to control the disease and the type of vaccines and vaccination schedule for ND vary depending on potential threat and virulence of the field virus. So, Various research has recommended that combination of live and inactivated ND vaccines administered simultaneously provide better protection and vaccine has been used successfully in control programmes in the area where there is intense poultry production [13]. Thermostable vaccine against Newcastle disease has been found successful in the Nigeria for controlling the disease [14]. Various vaccines are developed to prevent and control the disease. Mainly live vaccine are used for the Newcastle disease and there are four types of ND vaccine that are produced in the Nepal [15]. Vaccination when done correctly on the healthy birds prevents death and disease [16]. Mainly Heister and National Vaccine Production Lab produce vaccine in Nepal and many brand vaccine are also imported by large commercial farm from foreign country. Few vaccines and their vaccination protocol to prevent and control Newcastle disease are given below.

Table 2: ND vaccine available in Nepal

Name of Vaccine	Produced By	Type of Strain	Indication	Immunity	Vaccination Method	Transportation of Vaccine
RD F-1 Strain Vaccine	NVPL	Lentogenic	Vaccination of poultry for both meat and egg purpose	4 Weeks	Drinking water or skimmed milk, Intra ocular, Intra nasal	Transport in Icebox, Storage in deep fridge
Ranikhet R2B strain Vaccine	NVPL	Mesogenic	Vaccination of poultry for both meat and egg purpose	1 Year	Intramuscular	Transport in Cool box, Storage in deep fridge
RD La Sota Strain Vaccine	NVPL	Lentogenic	Vaccination of poultry for both meat and egg purpose	4 Weeks	Skimmed milk, Drinking water	Transport in Cool box, Storage in deep fridge
Newcastle disease Vaccine, La Sota Strain	Hester Biosciences Nepal Private limited	Lentogenic	Vaccination of chicken for both initial vaccination and revaccination	1 month	Intranasal, Intraocular, Drinking water, Spraying method	Transport in cool Box with temperature maintaining 2-8 degree celcius. Not to freeze.
ND I2 Strain Vaccine	NVPL	Avirulent	Vaccination of Backyard poultry	For 3 months	Intraocular	Vaccine is Heat resistant, Better to transport in Cool box

***Source:** National Vaccine Production Lab* [15]

Table 3: Vaccination schedule for broilers against newcastle disease

Age	Type of Vaccine	Vaccination method
5-7 days	F strain	Drinking water or Intra ocular
10-18 days	La Sota / F strain	Drinking water

Source: National Vaccine Production Lab [15]

Table 4: Vaccination schedule for layers against newcastle disease

Age	Type of Vaccine	Vaccination Method
5-7 days	F Strain	Intra Occular or Drinking water
28-30 days	La Sota or F Strain	Drinking Water
49-50 days	La Sota or F Strain	Drinking Water
8-10 Weeks	R2B Strain	Intramuscular
16 Weeks	La Sota or F Strain	Drinking Water

Source: National Vaccine Production Lab [15]

Status of ND vaccine production in Nepal

The vaccine against ND is produced in according to demand in the country. NVPL provide safe, potent and effective vaccine to the farmer which has passed all the quality standards according to OIE guidelines. The figure 5 describes the progress and target of ND vaccine production of different strains by NVPL in different years. The production of Ranikhet F1 is highest followed by Ranikhet R2B and Lasota and lowest production of I2 but NVPL is able to produce the required quantity of vaccine according to target and progress in production every year.

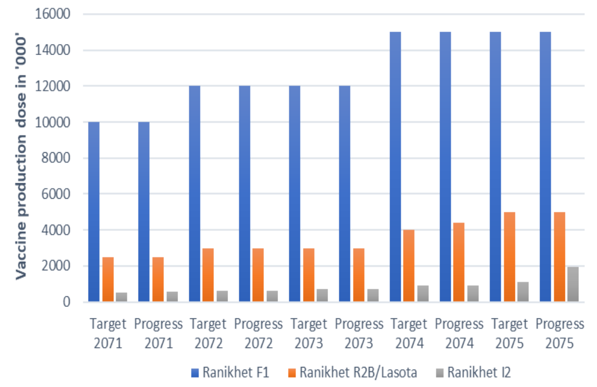

Figure 5: Status of Ranikhet (ND) vaccine produced by NVPL

Source: NVPL annual report 2075/76 [19]

Various factors influencing vaccination programs for control of Newcastle disease

Newcastle disease vaccination Programs have main goal of protecting birds from viral infection and clinical disease without causing any serious adverse vaccine reaction. There is no any thumb rule that all ND vaccination program will protect birds from infection. Various factors that directly influence vaccination programs and these factors must be considered during development of vaccination program to ensure maximum protection from disease. These factors may include virulence property of field challenge virus, potential exposure to virulent and pathogenic NDV, Virulence property of virus in vaccine to vaccinate the birds, age factor of the birds during the time of vaccination, genetic factors like genetic difference in birds, concurrent infection with various other avian pathogens that weakens the immune response, maternal antibody level in newly hatched chicks which can interfere with the vaccination [13]. The immunized birds can be clinically normal and still be infected and shed infecting ND virus. In California during 1971-1974, the outbreak of virulent ND occurs where vaccinated birds with ND vaccine became infected and shed virus for extended period of time [17]. The virulent ND virus was isolated from the embryonated eggs from clinically normal breeder hen which has high antibody titer to ND and clinically sick chick hatched from egg [18]. So, it is important to define goal for effectiveness of vaccination programs.

Future of Newcastle disease vaccine

Vaccine researcher and manufacturer should be focused on development of Ideal ND Vaccine. There is high demand for production of Ideal ND Vaccine. An Ideal ND vaccine should be heat stable, provide longer immunity, safe, cheap, easy to apply and should be able to prevent virus shedding and disease. Future research and study should be focus on development of potent vaccine against Newcastle Disease and prevent spread of disease and massive economic loss in Poultry industry.

Conclusion

In conclusion, Newcastle Disease is viral disease in the poultry which can be easily prevented and controlled by vaccination. A vaccination should be done according to proper vaccination schedule and proper route along with proper dose. Breakage of cold chain, improper site of vaccination, improper dilution may be the cause of vaccination failure. So, vaccination in proper way is a key factor for the prevention and control of the Newcastle Disease.

Abbreviation

Newcastle Disease (ND), Infectious Laryngotracheitis (ILT), Infectious Bursal Disease (IBD), National Vaccine Production Lab (NVPL), Avian Paramyxo Virus (APMV), Gross Domestic Product (GDP).

Authors' contribution

The manuscript composed by UP and co-author gives critical feedback on the manuscript before publication and all the author are agreed to final content for publication.

Acknowledgement

We are thankful to Dr. Saroj Chaudhary, Dr. Sonsy Dangol, Dr. Season Sainju and Dr. Shristi Shrestha for their special help and support.

References

1. FAO in Nepal. Retrieved from "http://www.fao.org/nepal/fao-in-nepal/nepal-at-a-, 2019. glance/en/"
2. Nepal Agritech. Retrieved From, 2018. "http://nepalpoultryandlivestock.com/testimonials/
3. Livestock Statistics of Nepal. Retrieved From https://www.moald.gov.np/publication/Agriculture%20Statistics, 2075.
4. Nepal commercial Poultry survey (2014/2015). Retrieved From "https: // kathmandupost.com/ money/ 2016/07/10/nepals-poultry-industry-worth-rs3372-billion"
5. Newcastle Disease in Poultry. Retrieved From: "https://www.msdvetmanual.com/poultry/newcastle-disease-and-other-paramyxovirus infections/newcastle-disease-in-poultry"
6. Van Boven M, Bouma A, Fabri TH, Katsma E, Hartog L, Koch G. Herd immunity to Newcastle disease virus in poultry by vaccination. Avian pathology. 2008; 37(1):1-5. DOI:"https://doi.org/10.1080/03079450701772391
7. Poultry Hub. Retrieved From, 2019. "http://www.poultryhub.org/health/disease/types- of-disease/newcastle-disease/"
8. Afonso CL, Miller PJ, Grund C, Koch G, Peeters B, Selleck PW, Srinivas GB. Newcastle disease (infection with Newcastle disease virus). Manual of diagnostic tests and vaccines for terrestrial animals, 7th ed. OIE, 2012, 555-574.
9. Miller PJ, Afonso CL, Spackman E, Scott MA, Pedersen, JC, Senne DA, Brown IH. Evidence for a new avian paramyxovirus serotype 10 detected in rockhopper penguins from the Falkland Islands. Journal of virology. 2010; 84(21):11496-11504. DOI: 10.1128/JVI.00822-10
10. Alexander DJ, Senne DA. Newcastle Disease and Other Avian Paramyxovirus. In: A Laboratory Manual for the Isolation, Identification and Characterization of Avian Pathogens, Dufour- Zavala L. (Editor in Chief) Swayne DE, Glisson JR, Jackwood MW, Pearson JE, Reed WM Woolcock PR. 4th ed., American Association of Avian Pathologists, Athens, GA, 2008, 135-141.
11. Veterinary Epidemiological Center, Annual Epidemiological Bulletin January-December 2018, Department of Livestock Service, Hariharbhawan, Lalitpur, 2018.
12. Gautam SP, Ghimire NP, Shrstha S, Gautam S. History of Vaccine Production and Vaccination in Nepal. Golden Jubilee Souvenir, Nepal Veterinary Association, 2018, 136-143.
13. Senne DA, King DJ, Kapczynski DR. Control of Newcastle Disease by Vaccination. Dev Biol (Basel). 2004; 119:165-170.
14. Nwanta JA, Abdu PA, Ezema WS. Epidemiology, challenges and prospects for control of Newcastle disease in village poultry in Nigeria. World's poultry science journal. 2008; 64(1):119-127. DOI: https://doi.org/10.1017/S0043933907001766
15. Hand book of Livestock and poultry vaccination. Vaccine and vaccination schedule of poultry, National vaccine production lab, 2018, 21-24.
16. Miller PJ, Afonso CL, El Attrache J, Dorsey KM, Courtney SC *et al*. Effects of Newcastle disease virus vaccine antibodies on the shedding and transmission of challenge viruses. Developmental & Comparative Immunology. 2013; 41(4):505-513.
17. Utterback WW, Schwartz JH. Epizootiology of velogenic viscerotropic Newcastle disease in southern California, 1971-1973. Journal of the American Veterinary Medical Association. 1973; 163(9):1080.
18. Capua I, Scacchia M, Toscani T, Caporale V. Unexpected isolation of virulent Newcastle disease virus from commercial embryonated fowls' eggs. Journal of Veterinary Medicine, Series B. 1993; 40(1-10):609-612.
19. Annual Report (2075/76) Retrieved from : http://nvpl.gov.np/downloadfile/Annual%20Report%2007576_1567339179.pdf

View publication stats
