# Supplementary material for: Newcastle disease burden in Nepal and efficacy of Tablet I2 vaccine in commercial and backyard poultry production
Source: PLoS One. 2023 Mar 10;18(3):e0280688. doi: 10.1371/journal.pone.0280688 (PMC10004539; doi:10.1371/journal.pone.0280688)
Supplement: Attachment — Submitted filename: comment.docx [file pone.0280688.s008.docx]

Authors have conducted ND and IAV prevalence study during 2018-19 by collecting samples from 40 commercial and 36 backyard poultry farms from the 10 districts of Nepal. An outbreak was also investigated. Biosamples such as Oral, cloacal and blood samples were pooled and tested for serological and molecular assessment of NDV and IAV. NDV and IAV were detected using multiplex PCR assay. Commercially available ELISA kits were used to detect and quantify ND and Influenza A Nucleoprotein. Three Genotypes I, II and VII c were identified. A thermostable I2 ND vaccine (Ranigoldunga) was developed taking master seed from the University of Queensland, Australia and its Tablet and Lyophilized formulations administered in drinking water and ocular application were evaluated in chicken (*Gallus domesticus*).

48 (Abstract):	The I2 Tablet ND vaccine showed more than 85% efficacy when administered either ocularly or in water, and has stability 50 of 30 days in room temperature.

Tablet form ND vaccine in water is missing in the trial?

141:	We collected additional samples from suspected unhealthy or sick birds from commercial (<5 birds) and backyard (< 2 birds) farms prior to our regular random sampling.

Analysis results may be incorporated.

170:	Questionnaire used for Biosecurity and biosafety risk assessment in poultry farms is missing?

181:	Mention about cloacal swabs in sample processing strategy.

347/358: Description on ELISA as appeared in table 5/6 may be placed in appropriate site.

401:	Commercial farms (n= 36) ? Needs correction

503:	Justification for variation may be incorporated with reference.

533 & 534:	Restructure the sentence to make more meaningful.

561:	Replace boiler to broiler

562:	More information is required about the vaccine already in use.

**General** :	Additional information on AIV genotyping and Phylogenetic analysis would have added been more informative. Prior to vaccination trial, 1% of the chickens were randomly selected, sampled and screened for NDV and IAV, including some water and feed samples. Results of water and feed samples are missing?

:
